# Improving replicability in single-cell RNA-Seq cell type discovery with Dune

**DOI:** 10.1186/s12859-024-05814-6

**Published:** 2024-05-24

**Authors:** Hector Roux de Bézieux, Kelly Street, Stephan Fischer, Koen Van den Berge, Rebecca Chance, Davide Risso, Jesse Gillis, John Ngai, Elizabeth Purdom, Sandrine Dudoit

**Affiliations:** 1 Department of Statistics, University of California, Berkeley, CA USA; 2 Division of Biostatistics, School of Public Health, University of California, Berkeley, CA USA; 3 Division of Biostatistics, Department of Population and Public Health Sciences, Keck School of Medicine, University of Southern California, Los Angeles, CA USA; 4 Cold Spring Harbor Laboratory, Cold Spring Harbor, NY USA; 5 Department of Applied Mathematics, Computer Science and Statistics, Ghent University, Ghent, Belgium; 6 Department of Molecular and Cell Biology, University of California, Berkeley, CA USA; 7 Department of Statistical Sciences, University of Padova, Padova, Italy; 8 Center for Computational Biology, University of California, Berkeley, CA USA

**Keywords:** Clustering, Single-cell, ScRNA-Seq, Consensus clustering, Replicability

## Abstract

**Background:**

Single-cell transcriptome sequencing (scRNA-Seq) has allowed new types of investigations at unprecedented levels of resolution. Among the primary goals of scRNA-Seq is the classification of cells into distinct types. Many approaches build on existing clustering literature to develop tools specific to single-cell. However, almost all of these methods rely on heuristics or user-supplied parameters to control the number of clusters. This affects both the resolution of the clusters within the original dataset as well as their replicability across datasets. While many recommendations exist, in general, there is little assurance that any given set of parameters will represent an optimal choice in the trade-off between cluster resolution and replicability. For instance, another set of parameters may result in more clusters that are also more replicable.

**Results:**

Here, we propose Dune, a new method for optimizing the trade-off between the resolution of the clusters and their replicability. Our method takes as input a set of clustering results—or partitions—on a single dataset and iteratively merges clusters within each partitions in order to maximize their concordance between partitions. As demonstrated on multiple datasets from different platforms, Dune outperforms existing techniques, that rely on hierarchical merging for reducing the number of clusters, in terms of replicability of the resultant merged clusters as well as concordance with ground truth. Dune is available as an R package on Bioconductor: https://www.bioconductor.org/packages/release/bioc/html/Dune.html.

**Conclusions:**

Cluster refinement by Dune helps improve the robustness of any clustering analysis and reduces the reliance on tuning parameters. This method provides an objective approach for borrowing information across multiple clusterings to generate replicable clusters most likely to represent common biological features across multiple datasets.

## Background

Improvements in single-cell RNA sequencing (scRNA-Seq) over the last decade have allowed the characterization of gene expression in collections of thousands to hundreds of thousands of cells. As datasets have grown in size by several orders of magnitude, cell type identification remains a primary step in data analysis [1]. We will focus here on the task of unsupervised clustering, which can be broadly defined as partitioning observations into clusters based on a set of features, without using any prior knowledge on the groupings. In the scRNA-Seq context, clustering aims to identify groups of cells that are defined by a unique and consistent transcriptomic signature. Such groups of cells can represent either transient features, such as cellular states in a developmental process, or more permanent features, such as cellular types.

Many clustering algorithms have been proposed for scRNA-Seq, most of which are adaptations from the clustering literature at large. Popular methods include SC3 [2], Seurat [3], and Monocle [4]. Duo et al. [5] offer a recent review of some scRNA-Seq clustering algorithms, identifying SC3 and Seurat as the best-performing methods across a wide range of benchmark settings. However, clustering remains a complex task. Kiselev et al. [6] outline the various challenges—both biological and computational—of this step, including technical noise, biological heterogeneity, and the impact of tuning parameters (or hyper-parameters) for the clustering algorithms. While some methods, including SC3, provide a way of selecting the optimal values of their main tuning parameters, most do not, leaving the choice to the user. Consensus clustering methods such as SC3 [2], scConsensus [7], and RSEC [8] try to bypass this issue, but they also rely on meta-parameters which can still have substantial impact on the results. Overall, replicating clustering results across datasets remains a difficult task. In this work, we deem clusters to be replicable if running the exact same clustering algorithm with the same tuning parameters on a related dataset yields similar clusters.

Additionally, the aforementioned clustering algorithms identify a pre-specified number of clusters either directly, as in *k*-means, or indirectly, through another tuning parameter. They implicitly assume that there is only one relevant level of clustering resolution, i.e., an optimal number of clusters, in the dataset. We argue that this is often not the case, since cell types usually have a hierarchy. For example, Tasic et al. [9] propose a tree structure for the mouse anterolateral motor (ALM) and primary visual (VISp) cortical areas. At the higher levels, cells can be clustered as neurons and non-neurons. Then, neurons can be further split into GABAergic and glutamatergic neurons and so on and so forth. This hierarchical structure means that the concept of an “optimal” number of clusters is not appropriate. Instead, many datasets can be better characterized by ever-finer levels of resolution. At the highest level, cells are grouped into broad clusters that are quite coarse, but are easily identifiable and very replicable across datasets. As the resolution increases, distinguishing between reproducible stable cell types and artifacts of the data becomes more challenging. Indeed, these clusters are more likely to reflect over-partitioning (cf. overfitting) of the data or the presence of transient states. This resolution-replicabilty trade-off is not obvious to quantify and is heavily dataset-dependent: it is not only influenced by the biological setting under study and its complexity, but also highly dependent on technical properties of the data, such as sequencing depth and number of cells [1].

By far the most common method to establish a hierarchy for pre-defined clusters is agglomerative hierarchical clustering, a bottom-up method in which clusters are merged one-by-one until they are all merged into a single cluster. This procedure yields a tree structure linking clusters that are merged together. The tree can also be defined by merging clusters according to the fraction of differentially expressed (DE) genes between them [8, 9]. While several extensive benchmarks of clustering methods have been proposed [5, 10], these only focus on the resulting partitions rather than the full hierarchical structure and generally assume that the correct number of clusters is known. Zappia and Oshlack [11] propose a representation of clustering trees to visually describe hierarchies, but this type of analysis relies heavily on user supervision.

Here, we present Dune, an ensemble method that aims to reconcile multiple clustering results and extract the common structure that they all capture. Dune relies on the assumption that, while different clustering algorithms run with different tuning parameters will naturally provide discrepant clusters, all good clustering methods should be able to identify a common higher-level clustering that is robust to the choice of tuning parameters. This represents a level of resolution that can be used with high confidence given the biology and the dataset’s characteristics. Dune identifies this common higher level of resolution shared by all methods without requiring any tuning by the user. Examining this level can both provide useful biological insight and help to compare various clustering methods.

In this manuscript, we first introduce the Dune algorithm. We then demonstrate that Dune outperforms agglomerative merging methods over a variety of simulation scenarios, as well as real scRNA-Seq and snRNA-Seq datasets from different sequencing platforms. We also discuss the value of Dune’s stopping point and assess Dune’s robustness and limitations.

**Fig. 1:**
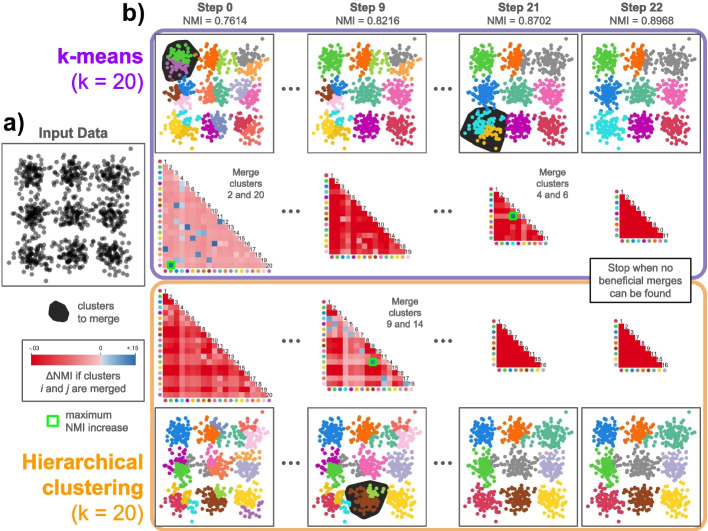
Overview of Dune ’s cluster merging strategy. Using a simulated 2-dimensional dataset, we show how Dune can take two naive clusterings as input and produce refined clusterings that are more appropriate to the structure of the data. **a** The simulated data were generated from 9 independent bivariate normal distributions, with centers arranged on a 3-by-3 grid, and 100 points per distribution. **b** The input data were clustered via *k*-means with $k = 20$ and via hierarchical clustering with $k = 20$, and the resulting partitions were provided to Dune for refinement. The initial agreement between the two partitions is measured by the normalized mutual information (NMI). Dune then evaluates all possible merges by searching over pairs of clusters within each partition to find the pair that will produce the largest increase in NMI when merged. After those clusters are merged, the process repeats. At each step, a single pair of clusters from either the *k*-means or hierarchical partition is merged until no beneficial merges can be found within either partition. This represents Dune’s natural stopping point and it produces clusters that are largely concordant between the two partitions and more accurately reflect the 9-cluster structure of the data

## Results

### Scope of Dune

We wish to delineate at the outset the scope of Dune, i.e., its underlying assumptions, its required inputs, and how to interpret and use its outputs. In practice, researchers often try multiple clustering algorithms to explore different aspects of their data (e.g., resolution levels) and assess robustness of their clustering results. Dune’s main assumption is that there is a common higher-level of clustering that should be identifiable by most decent clustering methods. Dune requires as input a set of clusterings, i.e., results from a variety of pre-processing steps, clustering algorithms, and associated tuning parameters applied to a given dataset, that all somewhat capture this higher level. It returns as output merged versions of each of these clusterings, obtained by producing hierarchies of clusters by merging clusters within each partition using information borrowed from the other partitions. As such, it is not a new clustering algorithm and it requires the user to make a number of subjective choices about both its input and its output. In particular, the user needs to select the set of input clusterings. They also need to select which of its outputs, i.e., which of the merged clusterings, to retain for downstream analysis. Figure 1 provides an illustrative example of Dune’s cluster refinement process.

As demonstrated in this manuscript, what Dune accomplishes, however, is to (1) improve upon each of the input clusterings (according to a wide-range of measures) and (2) lessen the impact of the choice of input clusterings and output clusterings on downstream analysis, by reducing the variability in the quality of the output compared to the input. In other words, the user is left to choose between improved clusterings and their choice is not as critical as if they were to select between the input clusterings.

In the following sections, we evaluate Dune and compare it to the two hierarchical tree merging methods, using five simulated datasets and four real datasets. We use the simulated datasets to investigate the value of Dune’s stopping rule. Then, we demonstrate the superiority of Dune on real datasets over a wide range of measures. Finally, we investigate the stability of the Dune algorithm to the clustering inputs and the sample size.

**Fig. 2:**
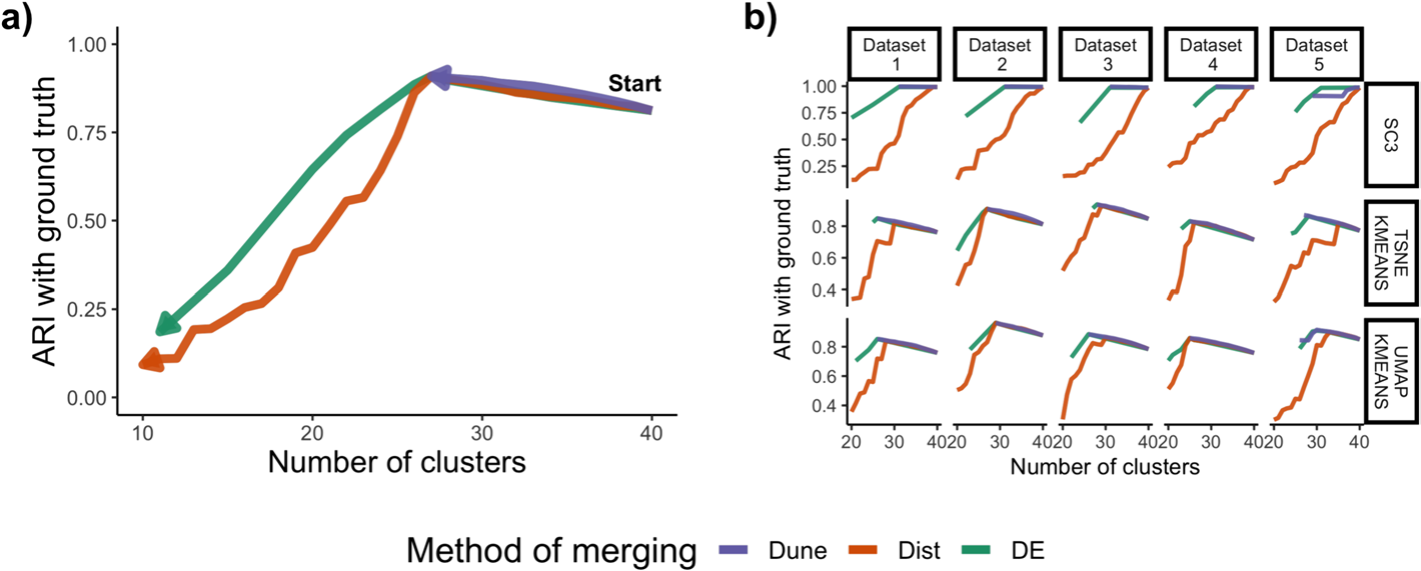
Dune stops at a meaningful level. Each of the three merging methods is applied to a simulated dataset and the Adjusted Rand Index (ARI) with the ground truth is tracked as the number of clusters decreases. **a** For Dataset 2 and tSNE+kMeans, Dune stops merging right where the concordance is maximal, while the other methods do not. **b** Over all clustering methods and datasets, Dune stops merging at one point, which always coincide with high agreement with the ground truth

### Dune has a natural stopping point

Unlike other merging methods, Dune provides a meaningful unsupervised stopping point: it merges clusters until no improvement in average Normalized Mutual Information (NMI) can be achieved. This stopping point identifies the level of resolution where all clusterings are close to full agreement. By contrast, the two hierarchical merging methods may continue to merge until there is only one cluster, which is not biologically meaningful or interesting. While various criteria for terminating these merging methods may be adopted and produce reasonable numbers of clusters, the resulting partitions can easily end up worse than the original inputs (Fig. 2a).

We investigate the stopping rule on simulated data. Using Splatter [12], we generated five simulated datasets of various complexity, each with 30 clusters. Overall, these datasets are simpler than in real settings. As such, methods such as Seurat and Monocle are able to assign cells to the correct clusters with close-to-perfect accuracy over a wide range of simulation parameters. To allow for merging, we therefore relied on simpler clustering methods, where we can specify the number of clusters and purposefully over-cluster. Following [5], we applied SC3 without the sc3_estimate_k function, as well as kMeans, using as input three-dimensional representations of the data obtained by running either t-SNE or UMAP on the normalized counts.

We compare Dune to two alternative cluster merging strategies, here called DE and Dist. Both alternative methods are based on a hierarchical organization of clusters, which we construct using Euclidean distances on the cluster medoids. The Dist method merges clusters according to this hierarchy and uses Dune’s stopping criterion, when the NMI between the input partitions is maximized. We note that this provides the Dist method with more information than it would otherwise have when merging clusters from a single partition. The DE method computes the percentage of differentially expressed genes between pairs of clusters and merges if this percentage fails to meet a certain threshold determined by an additional parameter (default = 0.05), as in the RSEC method [8]. See Methods 4.4 “Existing cluster merging methods” for more details.

In Fig. 2a, for example, we merged the clusters obtained from running tsne+kMeans on Dataset 2. For all three merging methods, as merging occurs, the resolution (i.e., number of clusters) decreases and the concordance with the ground truth increases at first, as measured with the adjusted Rand index (ARI, [13, 14]). Dune then stops when the agreement between cluster labels is at its peak. In this example, this coincides with the maximal agreement with the ground truth. On the other hand, DE and Dist keep on merging until there is only one cluster.

This result holds over all clustering methods and simulated datasets, as shown in Fig. 2b. Note that the DE method is at an advantage here since it relies on the statistical model used to simulate the counts in Splatter. On the other hand, Dune does not assume such a model, but performs on par with DE until Dune’s stopping point in 14 out of 15 cases. Using the NMI with ground truth instead of the ARI leads to similar results (see Fig. S7).

### Dune outperforms other methods on real datasets

We make use of four publicly available single-cell transcriptomics datasets to compare various cluster merging strategies. Two datasets, generated by the Allen Institute for Brain Science (AIBS) and originally described in [15], are comprised of cells from the mouse brain. These datasets make use of different sequencing technologies: the “AIBS scRNA” dataset uses single-cell RNA sequencing and the “AIBS snRNA” dataset uses single-nucleus RNA sequencing. Two more datasets, comprised of cells from human pancreatic samples, were generated by different labs and described in [16] and [17], respectively.

**Fig. 3:**
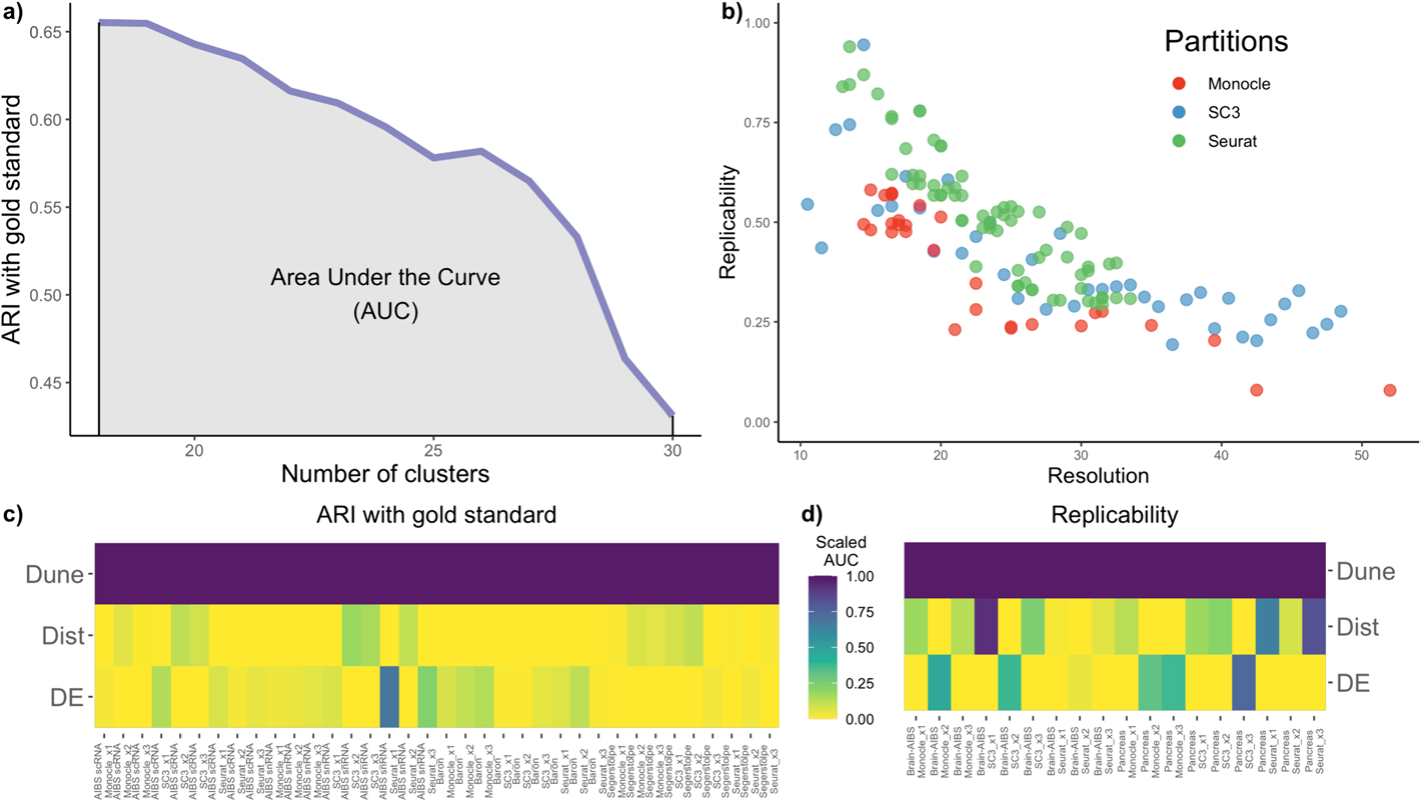
Comparison of methods. **a** SC3 was run on the AIBS mouse brain scRNA-Smart-Seq dataset for $\theta _{sc3}=0$ and merged with Dune (with $\theta _{Monocle}=45$ and $\theta _{Seurat}=1.2$, for Dune). The ARI with the labels from the original publication, treated as gold standard, was computed at each step of all three merging procedures. The area under this ARI curve was then computed. **b** SC3, Seurat, and Monocle were run on mouse brain datasets, for a wide range of tuning parameter values. Then, the MetaNeighbor method was used to find the clusters that are replicable between these two datasets and replicability was defined as the fraction of cells in replicable clusters. No “gold standard” partitions were used for replicability analysis. There is an apparent trade-off between resolution and replicability. **c** Repeating the procedure from **a** for three clustering methods, each with three different values of their respective tuning parameter $\theta$, and four datasets, yields 36 comparisons of AUC. The resulting 36 AUCs are displayed in a pseudocolor image, after being scaled to have a column min of zero and column max of 1. This was done to make AUC values comparable across datasets, clustering methods, and parameter values, since the AUC can have different scales across scenarios. **d** Similarly, replicability between datasets on the same biological system is tracked as clusters are merged and AUCs are computed. This yields 18 comparisons. The color legend is shared between both (**c**) and (**d**)

*Comparison with gold-standard clustering.* To evaluate Dune, we first considered how well the resulting merged clusters compare to the published labels. At each merge (i.e., iteration), we computed the ARI between the gold-standard and the merged clusters. This led to curves similar to those in Fig. 2a. The entire ARI curve can be summarized by computing the area under it, referred to herein as the area under the curve (AUC), as depicted in Fig. 3a. The AUC provides better stability relative to the final ARI and reflects the entire merging process rather than just the endpoint. This limits the impact of our choice of stopping criterion for the DE and Dist methods and reflects the difference between prioritizing merges based on a pre-determined hierarchy (as in DE and Dist) and based on maximizing between-partition NMI (as in Dune).

*Measuring clustering replicability across datasets.* We then considered the replicability of the clusters found by Dune compared to the other two merging strategies. We measured replicability by evaluating whether the method finds similar clusters for multiple independent datasets—for example, datasets on the same biological system but from different labs or technologies. We considered pairwise comparisons of the clusterings for each of the two mouse brain datasets and for each of the two human pancreas datasets. To measure replicability, we relied on the MetaNeighbor algorithm from [18], which identifies replicable clusters between pairs of datasets (see “Supplementary Methods, MetaNeighbor” for description). The replicability of a clustering was then defined as the fraction of cells in replicable clusters. We used this measure to compare Dune to other merging procedures. We note that this comparison does not rely on a “gold standard” partition; rather, the cluster merging strategies are evaluated solely on their ability to produce replicable clusters across different datasets on the same biological system.

*Illustration of the trade-off between cluster resolution and replicability.* Figure 3b displays replicability vs. resolution for a wide range of clustering results, where three clustering methods (SC3, Seurat, and Monocle) were run with a large grid of tuning parameter values, on the pair of mouse brain datasets. This clearly demonstrates the trade-off between replicability and resolution: as the number of clusters increases, the fraction of cells in replicable clusters decreases, regardless of the clustering method used. While the actual trade-off is specific to the biological context and the pair of datasets that are being considered, it should be stressed that a similar trade-off is clearly visible when applying the same type of analysis to the human pancreas datasets (Fig. S3). Note that although it might be tempting to use this figure to contrast and benchmark clustering methods, this would not be appropriate. Indeed, pre-processing steps were not identical between the three methods—as described in “Supplementary Methods, Data analysis”—and, as such, no direct comparison is possible.

As pairs of clusters are merged, the resolution decreases, so a well-performing merging method is one that improves the replicability of the clusters. Therefore, a natural way to benchmark merging methods is to measure how and if replicability improves as the number of clusters is reduced. Similar to the comparison with the gold-standard datasets, an area under the replicability curve can be computed to compare all three merging methods.

*Comparison of merging methods.* Figure 3c shows the results of benchmarking the three merging methods, Dune, DE, and Dist, using the ARI with respect to the gold-standard labels, over a multiplicity of scenarios. Dune and the other merging methods rely on one or multiple clustering results—in this work, clusterings from SC3, Seurat, and Monocle. Because each of these three clustering methods has tuning parameters than can affect its performance, we ran each method on a grid of tuning parameter values for each of the four datasets, as described in the “Methods, Data analysis” section. The AUC for the three merging methods across these 36 scenarios are displayed in Fig. 3c, with column-wise scaling to allow for easier display. Dune clearly outperforms the other two merging methods, ranking consistently first. For the replicability benchmark, since we considered pairs of datasets, the number of comparisons is halved. In Fig. 3d, Dune outperformed the other two merging methods in all 18 comparisons. Given these results, we forgo further comparisons and focus on Dune in the remainder of the manuscript.

### Dune increases the confidence of annotation

While cluster replicability is important in itself, producing robust and replicable clusters has implications for other biologically meaningful tasks, including cell type annotation. We investigated how Dune can be used to improve cell type annotation, which is a form of supervised classification, where labels learned on one dataset are used to annotate cells from another referred to as target dataset. Here, we relied on the annotation method of [3], since it also scores the confidence of annotation with a value between 0 and 1, with higher values corresponding to a more confident cell type assignment. We could therefore monitor how the average score among all cells of the target dataset evolved when using a clustering method before and after merging. Repeating this across all clustering methods, choices of tuning parameters, and reference datasets (more detail in the “Methods, cell type annotation” section) led to 36 scenarios. We found that merging with Dune consistently improved the confidence of the annotation: the average score increased by $20\%$ for the mouse brain datasets and $10\%$ for the pancreas datasets.

### Empirical robustness of Dune

Dune is a semi-supervised method in the sense that it still requires users to select its input: which and how many clustering methods to use, and with which tuning parameters. While Dune does not entirely alleviate the need to make these choices, it provides a higher level of robustness, compared to individual clustering methods. Using both the simulated and real datasets, we evaluated how much the output of Dune is impacted by upstream choices. As detailed below, Dune not only improves the overall quality of the individual clusterings, but, importantly, lessens the impact of the choice of input and output clusterings.

*Robustness to sample size.* Dune is very stable to downsampling. Decreasing the number of cells, either before clustering (Fig. S4a) or after clustering but before Dune (Fig. S5b), by up to $90\%$ has little negative effect on the quality of the merged cluster labels. For example, on simulated data, the ARI with the ground truth on a dataset with $n=500$ cells is never less than $97\%$ of its value for a dataset of $n=5000$ cells, as shown in Fig. S4a. Dune is also very stable to adding more clustering inputs. Using a variety of algorithms and associated tuning parameters as input to Dune on the simulated data, we can measure the impact of increasing the number of inputs from $R=2$ to $R=9$. For $R\ge 3$, increasing the number of of clustering inputs does not change the quality of the methods (Fig. S4c). Note, however, that computational times are increased, as Dune scales as $R^2$ (Section S-1.4). For this reason, we have found that in practice using $R=3$ inputs works best. More details on these evaluations can be found in the  “Methods, robustness” section and Section S-2.2.

Because Dune merges clusters, one might expect that small cell types would be lost, since they could easily be merged into larger ones. However, we find (see “Supplementary Results, rare cell types”) that Dune preserves those cell types $75\%$ of the time. The reverse is mostly linked to the quality of inputs.

*Robustness to quality of input clusterings.* While Dune is able to borrow information across multiple partitions, it does rely on the number and quality of the input clusterings. We find that the absolute number of input clusterings does not have a large effect on Dune’s cluster refinement process, except in the case of having only two initial partitions (Fig. S4c). Because these results are based on three basic clustering methods with various tuning parameter selections, they indicate the importance of diversity amongst the input clusterings, so that each may be able to find unique characteristics of the dataset.

As shown in Fig. S4b–c, the ranking of clusterings before merging is mostly conserved after merging, but the resolution of the initial clusterings does play a role in determining the final quality of the clusterings. Dune relies on merging to identify a common level of resolution, but if all input clusterings represent an under-partitioning of the data, little merging will be done. As such, inputs to Dune should err on the side of over-partitioning to allow merging to be effective and this should be taken into consideration when selecting tuning parameters. In general, we find that using Dune to refine a poor-quality partition might improve it enough to outperform a high-quality one without merging, but the high-quality partition with Dune merging will nearly always produce better results.

*Selection of output clustering.*

Finally, Dune merges clusters to improve concordance between its inputs, but it does not select one more preferably. At this point, as is the case with any clustering workflow, user intervention is needed to select which set of cluster labels to use for downstream analysis. Dune ensures that this step is more stable and less critical by increasing concordance between methods. Indeed, we can assess the variability in quality before and after merging, as measured by the ARI with the ground truth. Over all 53 simulations conducted, the variance in quality after merging with Dune is on average a third of the original variance, and is increased only in one case, when $n=100$.

Selecting the specific clustering output to retain is outside of the scope of Dune and other criteria need to be used. On simulated datasets, selecting the partition based on the average silhouette width leads to the best method $80\%$ of the time, as measured by ARI or NMI with the ground truth, and never leads to the worst. Likewise, on the mouse brain datasets, when evaluating with either replicability or ARI with the gold standard, selecting a clustering based on the average silhouette width leads to the best method $75\%$ of the time and the second best the remaining $25\%$. However, for the human pancreas datasets, the clustering with the highest average silhouette width has the lowest replicability and concordance with the gold-standard labels.

Overall, there is no single measure that will work all the time. Visual inspection or relying on external biological insight, such as known marker genes, is often the best guide. To demonstrate this, we provide a full workflow, explaining how to use Dune in practice to improve fully off-the-shelf clustering results and illustrating how to select the final output.

## Discussion and conclusions

We have introduced Dune, a new ensemble method which aggregates clustering results from multiple algorithms. Dune improves upon each of the input clusterings over a variety of measures and, in particular, can correctly navigate the resolution-replicability trade-off in cluster analysis. In this regard, Dune outperforms more commonly used hierarchical merging methods. We stress that Dune is not a new clustering algorithm; instead, it relies on different clustering methods to identify the highest resolution at which cluster quality (i.e., replicability across datasets) remains high. In doing so, Dune identifies the commonalities of the input clusterings and uses them to improve each of these clusterings individually. It also lessens the impact of the choice of input clusterings and output clusterings on downstream analysis, by reducing the variability in the quality of the output compared to the input. That is, the user is left to choose between improved output clusterings and their choice is not as critical as if they were to select between the input clusterings. Furthermore, as a result of merging clusters, Dune provides a sensible hierarchy on the clusters based on their commonality across different methods. As we go up in this hierarchy, the number of clusters is reduced, but their replicability improves.

Dune automatically stops at a meaningful resolution level, where all clustering algorithms are in close agreement, while the other methods either keep merging until all clusters are merged into one or require user supervision to stop early. This feature helps users in identifying reliable structure in their scRNA, snRNA, or similar datasets. In contrast, the manual choice of a stopping point is difficult since, in practice, it is often impossible to measure replicability given the lack of a second appropriate dataset.

We focused on the normalized mutual information (NMI) to decide which clusters to merge. The current implementation of Dune also allows users select the ARI as merging criterion; other merging criteria could be implemented. Dune also allows for some cells to remain unclustered, such as currently implemented in RSEC [8]. Possible extensions include clustering methods that do not cluster all cells unambiguously, e.g., soft or fuzzy clustering methods which may assign some cells to multiple clusters based on weights. For now, using such methods as input to Dune would require forcing hard assignments of the cells to clusters (possibly to their nearest cluster). Extensions of the NMI to fuzzy clustering have been proposed [19] and could be evaluated.

One limitation of the current study is its reliance on previously published clusters. When evaluating cluster merging strategies on a single dataset, we treat the original authors’ clustering as a “gold standard.” We implicitly assume that these partitions represent some level of meaningful biological signal, as the original publications were concerned with biological mechanisms and contained varying degrees of external validation for their findings [15–17]. Similarly, when evaluating replicability across comparable datasets, we rely on the veracity of the results produced by the MetaNeighbor algorithm [18].

This manuscript concerns the question of unsupervised clustering. Recent work in supervised clustering [20–23] has proposed labeling cells in a new dataset by relying on information contained in other datasets or even cell atlases. In practice, these methods define marker genes for known cell types and build classifiers to assign new cells to these cell types. In particular, Garnett [24] allows a hierarchical clustering structure, but one that needs to be predefined, and scClassify [25] uses the HOPACH [26] algorithm to establish a hierarchy in the training dataset. Most of these algorithms can also identify new cell types not present in the reference. It is therefore possible to use Dune in a supervised clustering context, where one first identifies the cells that have known cell types and, if these do not provide information to help cluster the rest of the cells, one removes them and applies unsupervised clustering methods and Dune to the remaining cells.

While Dune has only been benchmarked on scRNA-Seq and snRNA-Seq datasets, it is a general framework that can be applied in any clustering setting.

## Methods

### Clustering setup and comparison

Consider a—possibly high-dimensional—dataset of *n* observations, $\mathbf{X} = \{x_1, \ldots , x_n\}$, where $x_i \in R^J$, $i=1,\ldots , n$. For instance, in scRNA-Seq, $x_i$ can correspond to the gene expression measures (i.e., normalized read counts) or to the reduced-dimension coordinates of cell *i*. Represent the results of any (non-fuzzy) clustering method as a partition, $\mathbf{P}$, which splits the set of *n* observations into *k* disjoint subsets or clusters, $\{{\mathcal{C}}_1,\ldots ,{\mathcal{C}}_k\}$, where: (1) ${\mathcal{C}}_i \cap {\mathcal{C}}_j = \emptyset$, $\forall i\ne j \in \{1,\dots ,k\}$, and (2) $\cup _{i \in \{1,\dots ,k\}} {\mathcal{C}}_i = \mathbf{X}$.

**Fig. 4:**
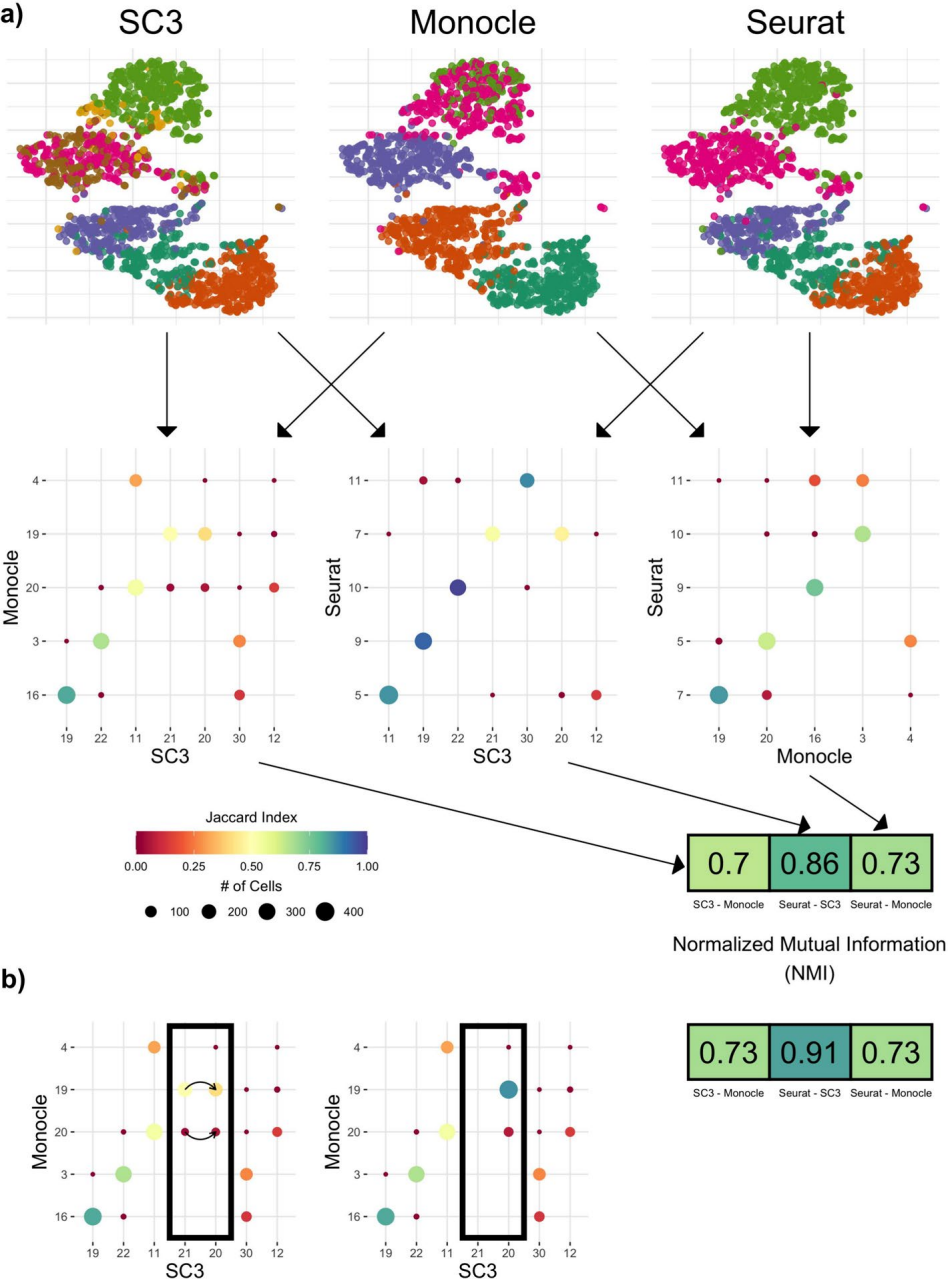
Measuring and improving the concordance between clusterings. We used a subset of the AIBS mouse brain snRNA Smart-Seq dataset [15] as an example. **a** SC3, Monocle, and Seurat were run on the dataset and their results are displayed using scatterplots of the first two t-SNE components, where the color of the plotting symbol corresponds to the cluster label. Each pair of clusterings was then compared using a confusion matrix, resulting in three such matrices. For a pair of clusterings/partitions, a confusion matrix is a contingency table, where each entry corresponds to the number of observations in both a cluster from the first partition and a cluster from the second. The size of the dot represents the number of observations in both clusters and the color corresponds to the Jaccard index. Each confusion matrix produces one NMI value. **b** Merging Clusters 20 and 21 from SC3 into one cluster changes the confusion matrix and increases the NMI

Accordingly, a collection of *R* clustering results may be represented as multiple partitions, $\mathbf{P}_1, \ldots , \mathbf{P}_R$, with partition $\mathbf{P}_r$ containing $k_r$ clusters, $r=1,\dots , R$. This set of *R* clustering results is generally produced by running *R* clustering algorithms (or the same algorithm with different tuning parameter values) on the same dataset. An example can be seen in Fig. 4a, where a small subset of the AIBS mouse brain snRNA Smart-Seq dataset [15] (see the “Methods, Case Studies” section) is used to demonstrate some of the main concepts underlying Dune. The first row displays three examples of clusterings (i.e., sets of cluster labels) produced by three different clustering algorithms applied to the same dataset, reduced to two dimensions using t-distributed stochastic neighbor embedding (t-SNE) [27–29]. All three methods identify similar—but not identical—clusters. Indeed, the algorithms output partitions with different levels of resolution. For example, Monocle splits the bottom region (on the t-SNE plot) into two clusters, while the other two methods find three clusters. Likewise, Monocle and SC3 find two clusters in the top region, while Seurat only finds one.

**Fig. 5:**
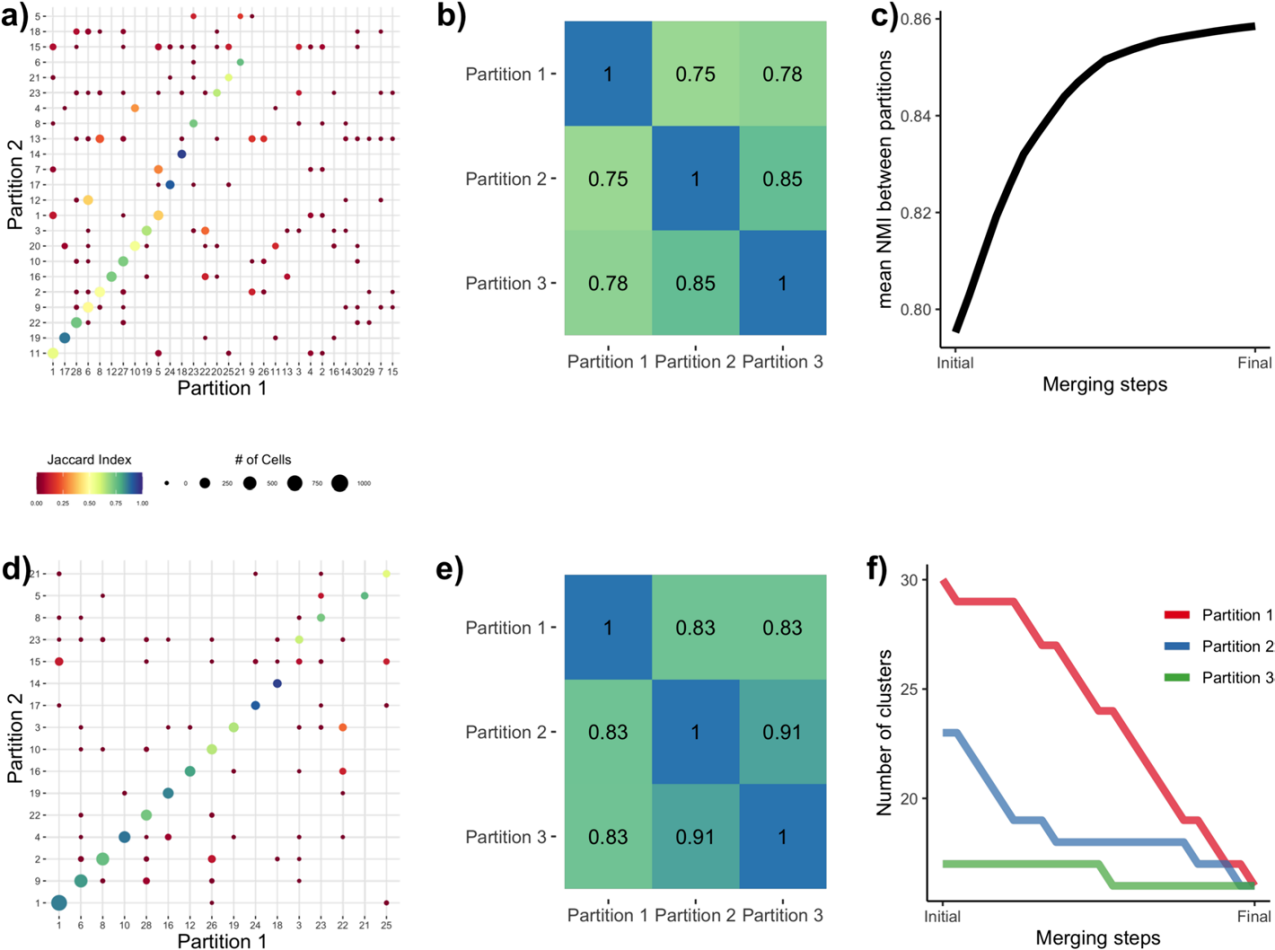
Illustrating Dune on a dataset with three sets of cluster labels. We used the AIBS mouse brain scRNA Smart-Seq dataset [15] as an example. Before any merging, the sets of cluster labels—or partitions—resulting from running SC3, Seurat, and Monocle have a moderate agreement. **a** displays the confusion matrix between two of the partitions, where each entry corresponds to the number of observations in both a cluster from Partition 1 and a cluster from Partition 2. The confusion matrix shows that while many cells are similarly clustered in the two partitions, i.e., along the main diagonal, many others are not. This can be summarized by the NMI between Partitions 1 and 2. **b** displays a pseudocolor image of the matrix of all pairwise NMIs between the three partitions. **c** illustrates that the average NMI between partitions increases as pairs of clusters are merged when applying Dune. After running Dune, the confusion matrix in (**d**) and the pairwise NMI matrix in **e** both show that the partitions are indeed more similar. **f** shows that, at each merging step, the number of clusters in one of the partitions is decreased by one, in Dune’s greedy procedure to improve the average NMI by merging pairs of clusters

The discrete joint distribution for two partitions of a dataset can be defined using a contingency table (see Table S1). Examples of contingency tables can be found in Figs. 4a, b, 5a, d, where the overlap between two clusters from any pair of clusterings is displayed both in terms of the number of cells in the intersection, and the Jaccard index (i.e., the cardinality of the intersection of the two clusters over the cardinality of their union; [30]). Rows and columns are ordered so as to maximize, as much as possible, the sum of the diagonal entries.

Contingency tables, also known as confusion matrices, can be further summarized using the normalized mutual information (NMI). The NMI is defined thus,

1 $$\begin{aligned} \text {NMI}(\mathbf{P}_1, \mathbf{P}_2) = \frac{2\times ( \mathbf{H}(\mathbf{P}_2) - \mathbf{H}(\mathbf{P}_2|\mathbf{P}_1))}{\mathbf{H}(\mathbf{P}_1) + \mathbf{H}(\mathbf{P}_2)}, \end{aligned}$$

where $\mathbf{H}$ is the entropy function (see the “Supplementary Methods, NMI” section for more details). The NMI is a commonly-used measure for the agreement between two sets of clustering labels. As can be seen in the confusion matrices, SC3 and Seurat have the highest level of agreement. Indeed, this is also reflected in the fact that they have the highest NMI of any pair.

### Dune with NMI merging

Given *R* partitions, $\mathbf{P}_1,\ldots ,\mathbf{P}_R$, with $\mathbf{P}_r$ containing $k_r$ clusters ($r=1,\ldots , R$), Dune seeks to improve the overall agreement among these, as measured by the average NMI over all pairs of partitions, through an iterative process of merging clusters within partitions. An example of the merging is displayed in Fig. 4b. Clusters 20 and 21 from SC3 are merged together, resulting in one larger cluster (named 20). Doing so increases the agreement between SC3 and Monocle in the confusion matrix, as reflected by an increase in NMI from 0.7 to 0.73. This merge also improves the NMI between SC3 and Seurat (from 0.86 to 0.91) and hence increases the overall agreement among the three clusterings. This is the main idea behind Dune.

Specifically, Dune searches over each partition $\mathbf{P}_r$ and over each of $$\left( {\begin{array}{c}k_r\\ 2\end{array}}\right)$$ pairs of clusters in $\mathbf{P}_r$ for the pair which produces the largest improvement in NMI when merged, i.e.,

2 $$\begin{aligned} (r^*,i^*,j^*):=\mathop {\mathrm{arg\,max}}\limits _{\begin{array}{c} r \in \{1,\dots ,R\} \\ i,j \in \{1,\dots ,k_r\} \end{array}} \sum _{\{s: s \in \{1,\dots ,R \}, s \ne r \}} \text {NMI}(\mathbf{P}_r^{i \cup j}, \mathbf{P}_s) - \text {NMI}(\mathbf{P}_r, \mathbf{P}_s), \end{aligned}$$

where $\mathbf{P}_r^{i \cup j}$ is the partition created by merging clusters ${\mathcal{C}}^r_i$ and ${\mathcal{C}}^r_j$ in partition $\mathbf{P}_r$.

Thus, the Dune algorithm can be viewed as an iterative algorithm for maximizing the average pairwise NMI of a collection of clustering results. Note that the NMI is only one of a variety of criteria that could be used to guide merging. The current implementation of Dune is flexible and allows for other measures. In particular, all benchmarks have also been conducted using the adjusted Rand index or ARI [13, 14], see Sections S-1.3 and S-2.3.

Dune amounts to a greedy algorithm for maximizing the average NMI: at each step, we find the pair of clusters that, when merged, lead to the greatest improvement in average NMI. Once we have identified this pair of clusters, we update the collection of partitions. We continue iterating until no beneficial merge can be identified, that is, we stop updating when

$$\begin{aligned} \max _{r,i,j} \sum _{s \ne r} \text {NMI}(\mathbf{P}_r^{i \cup j}, \mathbf{P}_s) - \text {NMI}(\mathbf{P}_r, \mathbf{P}_s) < 0. \end{aligned}$$

This greedy approach means that each update step is constrained to merging a single pair of clusters from a single partition. As such, we never merge three clusters together in one iteration or two pairs of clusters in the same or in separate partitions. Therefore, in all our simulations and case studies, we never converge to the naive optimal solution of merging all clusters, which does represent a full agreement between the partitions but is of no practical interest. We discuss in greater detail Dune’s greedy search strategy in the “Supplementary Methods, Scaling” section.

While Dune provides a natural stopping point for merging, it is also possible to stop earlier in the merging process, by tuning the merging parameter $m_{\textsf {Dune}}$, which is defined as the fraction of NMI improvement over the total NMI improvement when using Dune’s natural stopping point. For example, $m_{\textsf {Dune}} =.5$ means that Dune returns the merged partitions that have an average NMI halfway between the average NMI of the original partitions and the mean NMI of the final ones.

We demonstrate how the Dune algorithm works in Fig. 5, using the AIBS mouse brain scRNA Smart-Seq dataset, a scRNA-Seq dataset of 6,300 mouse brain cells described in the “Methods, Case Studies” section. For this example, we ran SC3, Seurat, and Monocle to obtain our initial clustering results for input into Dune ($R=3$). Figure 5a displays the confusion matrix for a pair of clusterings (SC3 and Monocle) before any merging and Fig. 5b displays a pseudocolor image of the matrix of all pairwise NMIs for the three clusterings before any merging. The agreement between the three methods is moderate. Indeed, the pairwise NMIs vary between 0.75 and 0.85 in Fig. 5b. However, as can be seen in the confusion matrix, the clusterings do capture a shared underlying structure, which will serve as grounding for the Dune merging. Figure 5d shows the confusion matrix for the same two partitions as in 5a, after merging with Dune. We can see that we have, by design, fewer clusters in both partitions, but also that the concordance between the two partitions is greatly improved (as indicated by the color of the plotting symbols, which represents the Jaccard index). This is further evidenced in Fig. 5e, where the pairwise NMIs between the three partitions are displayed. The average NMI after all merging steps increased from 0.79 to 0.86. Figures 5c and f demonstrate the evolution of the average NMI and of the number of clusters per partition through the Dune merging process. At each step, we merge the pair of clusters that leads to the greatest increase in average NMI. Hence, at each step, the average NMI increases (Fig. 5c) and the number of clusters in one of the partitions decreases by one (Fig. 5f). The final partitions are achieved when the average NMI can no longer be improved.

### Software implementation and run time

The Dune algorithm is implemented in an open-source R package released through the Bioconductor Project (https://bioconductor.org/packages/release/bioc/html/Dune.html). It is implemented in a fully-parallel and efficient manner. Run time for a large dataset of $\sim 130,000$ cells, with 3 partitions of respectively 100, 68 and 45 clusters, is under 15 min with 10 CPUs. The package also contains plotting functions, which were used to create many of the figures in the present paper and provide options to create GIFs and track the evolution of the average NMI or confusion matrices over the merging steps.

### Existing cluster merging methods

Once a set of clusters has been identified, one can build a hierarchical tree for these clusters and then merge clusters that are similar. This involves specifying a measure of distance or similarity between individual observations (i.e., cells) as well as between clusters. It should be noted that the distance used to build the tree of clusters need not be the same as the distance used to merge clusters.

For scRNA-Seq datasets, commonly-used between-cell distance measures include the Euclidean distance and one minus the Spearman correlation coefficient. Between-cluster distances include classical linkage measures used in hierarchical clustering, e.g., maximum/minimum/average of all pairwise distances between observations in two clusters or distance between the cluster averages or medoids. For scRNA-Seq, another sensible between-cluster similarity measure is the proportion of differentially expressed (DE) genes between clusters [8, 9]. A detailed discussion of such measures is out of the scope of this manuscript [31].

Here, we consider two possible merging approaches. In both cases, we compute the cluster medoids (median of observations within the cluster) based on the log-transformed count matrix (adding 1 to avoid taking the log of zero). We then build a hierarchical tree of clusters using the Euclidean distance between the cluster medoids. The first merging approach directly uses this tree to decide how to merge clusters. Specifically, clusters are merged bottom-up, starting with the two clusters that are closest in the tree, and then iteratively until all clusters are merged. The parameter $m_{Dist} = n_{merges}$, the number of merges (between 0 and the initial number of clusters minus one), controls the amount of merging. The second approach follows the method implemented in RSEC. It computes the percentage of DE genes between clusters, using the limma package [32] ($LIMMA, RRID:SCR\_010943$), where a gene is declared DE if its nominal FDR adjusted *p*-value is below 0.05 [33]. The main tunable parameter is $m_{DE} = \alpha \in [0, 1]$, the threshold for the percentage of DE genes below which we merge. We name these two methods Dist and DE, respectively.

### Simulation study

*Simulation study design.* To generate simulated datasets with known ground truth, we relied on the Splatter package [12]. Datasets of $n=5000$ cells and $J=10^4$ genes were generated with 30 cell types. The average proportion of differentially expressed genes between clusters, DE, was tuned between datasets. Datasets are numbered in order of decreasing separation between clusters, from 1 to 5, denoting increasing complexity. More details are given in the ‘Supplementary Methods, Simulations‘ section. Note that the simulation framework is built on a similar statistical model as the DE method and therefore provides it with an a priori advantage.

*Data analysis.*

Outlier genes and cells were removed and the data were normalized using the Seurat workflow. Two-dimensional uniform manifold approximation and projection (UMAP) plots of each dataset can be found in the supplementary Figures S2a–e. The *k*-means clustering algorithm was run with $k=40$ on reduced-dimensional coordinates from either t-SNE or UMAP. SC3 was also run with $k=40$. Then, clusters were merged using either Dune (with ARI or NMI as merging criterion), the DE, or Dist methods.

### Case studies

*AIBS Smart-Seq mouse brain datasets.* We used the two AIBS mouse brain Smart-Seq datasets produced as part of the Brain Initiative Cell Census Network (BICCN; $RRID:SCR\_015820$) and described in [15]; one corresponds to single-cell sequencing (*Zeng sc SSv4*, 6300 cells) and the other to single-nucleus sequencing (*Zeng sn SSv4*, 6278 cells). We use the original publication’s subclass labels as gold-standard cluster labels for these datasets, which were obtained using the iterative hicat method from [9] and then manually annotated and produced respectively 17 and 19 clusters. The datasets can be downloaded from the Neuroscience Multi-omics Archive ($RRID:SCR\_002001$; nemoarchive.org). More details on the parent dataset (https://assets.nemoarchive.org/dat-ch1nqb7) and data access can be found in [15].

*Human pancreas datasets.* We focus on two datasets from [16] (8568 cells) and [17] (3514 cells), which we name *Baron* and *Segerstople*, respectively. Both datasets were downloaded from https://hemberg-lab.github.io/scRNA.seq.datasets/ on October $1^{\text {st}}$, 2018. We use the clusters from the original publications as gold-standard clusters. In *Baron*, cells are clustered using hierarchical clustering with a final manual merging step, producing 14 clusters. In *Segerstople*, cells were assigned to manually-defined clusters using prior biological knowledge, producing 14 clusters.

*Pre-processing.* Details on pre-processing are given in the ‘Supplementary Methods, Data Analysis” section. For each of the datasets, we ran $R=3$ popular [1] clustering methods: SC3 (version 1.18.0), Seurat (version 3.1.3), and Monocle (monocle3 version 0.2.2), with various tuning parameters. The first two have been consistently ranked as some of the best-performing clustering algorithms in benchmark studies [5, 10], while the last relies on the same clustering algorithm as Seurat, but with different pre-processing choices and parameter tuning.

### Supplementary Information

Supplementary file 1

## Data Availability

The Pancreas datasets were downloaded from the Hemberg group website, https://hemberg-lab.github.io/scRNA.seq.datasets/human/pancreas/, on October 1st, 2018. The AIBS datasets can be obtained from the Neuroscience Multi-omics Archive (***RRID*** : ***SCR*****_002001**; nemoarchive.org), *Zeng sn SSv4* at https://assets.nemoarchive.org/dat-k7p82j4 and *Zeng sc SSv4* at https://assets.nemoarchive.org/dat-55mowp9. The results from this paper can be reproduced using code from the following GitHub repository: https://github.com/HectorRDB/Dune_Paper. The Dune method is implemented in an open-source R package released through the Bioconductor Project (http://www.bioconductor.org/packages/release/bioc/html/Dune.html).
