# Supplementary material for: Improving replicability in single-cell RNA-Seq cell type discovery with Dune
Source: BMC Bioinformatics. 2024 May 24;25:198. doi: 10.1186/s12859-024-05814-6 (PMC11127396; doi:10.1186/s12859-024-05814-6)
Supplement: Supplementary file 1 [file 12859_2024_5814_MOESM1_ESM.pdf]

Supplementary Material

S-1 Supplementary methods

S-1.1 Notation

Following the "Methods" section, we consider a – possibly high-dimensional – dataset of n observations, $\mathbf{X} = \{x_1, \dots, x_n\}$, where $x_i \in R^J$, $i = 1, \dots, n$. For instance, in scRNA-Seq, x_i corresponds to the J gene expression measures (i.e., normalized read counts) of cell i. Represent the results of any (non-fuzzy) clustering method as a partition, $\mathbf{P}$, which splits the set of n observations into k disjoint subsets or clusters, $\{\mathcal{C}_1, \dots, \mathcal{C}_k\}$, where: **1)** $\mathcal{C}_i \cap \mathcal{C}_j = \emptyset$, $\forall i \neq j \in \{1, \dots, k\}$, and **2)** $\cup_{i \in \{1,\dots,k\}} \mathcal{C}_i = \mathbf{X}$. Accordingly, a collection of R clustering results may be represented as multiple partitions, $\mathbf{P}_1, \dots, \mathbf{P}_R$, with partition $\mathbf{P}_r$ containing k_r clusters, $r = 1, \dots, R$. For each observation x_i, denote by $c_{i,r} \in \{\mathcal{C}_1^r, \dots, \mathcal{C}_{k_r}^r\}$ the cluster to which it belongs in partition $\mathbf{P}_r$. We also let $K = \max_r\{k_r\}$, i.e., the largest number of clusters among all partitions.

S-1.2 Normalized mutual information

Consider two partitions of a dataset, $\mathbf{P}_1$ and $\mathbf{P}_2$. These specify a discrete joint distribution defined by the contingency table with $(i,j)^{\text{th}}$ entry $n_{i,j}$ equal to the number of observations both in cluster i of partition $\mathbf{P}_1$ and cluster j of partition $\mathbf{P}_2$ (Table S1). Examples of contingency tables between two partitions can be found in Figures 4a, 4b, 5a, and 5d.

Table S1: *Contingency table for two partitions* $\mathbf{P}_1$ *and* $\mathbf{P}_2$*.*

	$\mathcal{C}_1^2$	$\mathcal{C}_2^2$	$\dots$	$\mathcal{C}_{k_2}^2$	Sums
$\mathcal{C}_1^1$	$n_{1,1}$	$n_{1,2}$	$\dots$	n_{1,k_2}	a_1
$\mathcal{C}_2^1$	$n_{2,1}$	$n_{2,2}$	$\dots$	n_{2,k_2}	a_2
$\vdots$	$\vdots$	$\vdots$	$\ddots$	$\vdots$	$\vdots$
$\mathcal{C}_{k_1}^1$	$n_{k_1,1}$	$n_{k_1,2}$	$\dots$	n_{k_1,k_2}	a_{k_1}
Sums	b_1	b_2	$\dots$	b_{k_2}	

In general, the mutual information [13] of two random variables measures their concordance. For partitions $\mathbf{P}_1$ and $\mathbf{P}_2$, the mutual information is defined as

$$\begin{aligned}\mathbf{I}(\mathbf{P}_1, \mathbf{P}_2) =& \mathbf{H}(\mathbf{P}_1) - \mathbf{H}(\mathbf{P}_1|\mathbf{P}_2)\\ =& \mathbf{H}(\mathbf{P}_2) - \mathbf{H}(\mathbf{P}_2|\mathbf{P}_1),\end{aligned}$$

where $\mathbf{H}$ is the entropy function. In terms of the contingency table notation, the mutual information is computed as

$$\mathbf{I}(\mathbf{P}_1, \mathbf{P}_2) = -\sum_{i=1}^{k_1} a_i \log(a_i) + \sum_{i=1}^{k_1}\sum_{j=1}^{k_2} n_{i,j} \log\big(\frac{n_{i,j}}{b_j}\big). \tag{3}$$

The mutual information can be normalized to yield a value between zero and one. While several variants exist, we selected the following definition for the normalized mutual information (NMI)

$$\mathrm{NMI}(\mathbf{P}_1, \mathbf{P}_2) = \frac{2 \times \mathbf{I}(\mathbf{P}_1, \mathbf{P}_2)}{\mathbf{H}(\mathbf{P}_1) + \mathbf{H}(\mathbf{P}_2)}. \tag{4}$$

For R partitions, the overall level of concordance can be quantified by the average NMI for all possible pairs of partitions

$$\overline{\mathrm{NMI}}(\mathbf{P}_1, \dots, \mathbf{P}_R) = \frac{1}{\binom{R}{2}} \sum_{\{(r,s):r,s\in\{1,\dots,R\},r<s\}} \mathrm{NMI}(\mathbf{P}_r, \mathbf{P}_s). \tag{5}$$

Note that, in the case of $R = 2$ partitions, this is simply the NMI between the two partitions. If one considers the matrix of pairwise NMIs between partitions, such as displayed in Figures 5b and e, then the average NMI is defined as the mean of the upper(or lower)-triangular matrix.

S-1.3 Adjusted Rand index

The Rand index [13] measures the concordance between two partitions, $\mathbf{P}_1$ and $\mathbf{P}_2$. Denote by $a = |\{(x_i, x_j) \in \mathbf{X}^2 : (c_{i,1} = c_{j,1})\&(c_{i,2} = c_{j,2})\}|$ the number of pairs of observations that are in the same cluster for both partitions $\mathbf{P}_1$ and $\mathbf{P}_2$ and by $b = |\{(x_i, x_j) \in \mathbf{X}^2 : (c_{i,1} \neq c_{j,1})\&(c_{i,2} \neq c_{j,2})\}|$

the number of pairs of observations that are in different clusters for both partitions $\mathbf{P}_1$ and $\mathbf{P}_2$. The Rand index is then the ratio of $a+b$ over the total number of pairs of observations

$$\mathrm{RI}(\mathbf{P}_1, \mathbf{P}_2) = \frac{a+b}{\binom{n}{2}} \in [0,1]. \tag{6}$$

Thus, intuitively, the Rand index is the proportion of pairs of observations for which the two partitions are in agreement.

However, the Rand index does not account for the fact that a pair of observations might be in the same (different) cluster(s) in the two partitions purely by chance. The adjusted Rand index (ARI) [14] adjusts for the level of concordance expected by chance, yielding a value between -1 and $+1$. Specifically, considering $\mathbf{P}$ a fixed partition and R a random permutation of $\mathbf{P}$, then $\mathbb{E}[\mathrm{ARI}(\mathbf{P}, R)] = 0$, where the expected value is over all cluster permutations (i.e., permutations of the cluster assignments of the observations, while keeping the number of clusters and the sizes of the clusters fixed). Negative values indicate less than the expected level of concordance and positive values indicate more than the expected level of concordance. The ARI relies on the contingency table of two partitions $\mathbf{P}_1$ and $\mathbf{P}_2$, with the $(i,j)^{\mathrm{th}}$ entry $n_{i,j}$ defined as the number of observations both in cluster i of partition $\mathbf{P}_1$ and cluster j of partition $\mathbf{P}_2$ (Table S1). Given the contingency table notation, the adjusted Rand index is defined as

$$\mathrm{ARI}(\mathbf{P}_1, \mathbf{P}_2) = \frac{\sum_{i,j}\binom{n_{i,j}}{2} - \frac{1}{\binom{n}{2}}\sum_i\binom{a_i}{2}\sum_j\binom{b_j}{2}}{\frac{1}{2}\left(\sum_i\binom{a_i}{2} + \sum_j\binom{b_j}{2}\right) - \frac{1}{\binom{n}{2}}\sum_i\binom{a_i}{2}\sum_j\binom{b_j}{2}}. \tag{7}$$

For R partitions, the level of concordance can be quantified by the average ARI for all possible pairs of partitions

$$\overline{\mathrm{ARI}}(\mathbf{P}_1, \ldots, \mathbf{P}_R) = \frac{1}{\binom{R}{2}} \sum_{\{(r,s):r,s\in\{1,\ldots,R\},r<s\}} \mathrm{ARI}(\mathbf{P}_r, \mathbf{P}_s). \tag{8}$$

Note that, in the case of $R = 2$ partitions, this is simply the ARI between the two partitions. If one considers the matrix of pairwise ARIs between partitions, such as displayed in Figures 5b and e, then the average ARI is defined as the mean of the upper(or lower)-triangular matrix.

S-1.4 Dune's search strategy

Computational scalability using contingency tables. Computing contingency tables for n pairs of labels scales as $O(n)$, since each observation must be assigned to a cell of the table. For large datasets, computing contingency tables for all pairs of cluster labels over all possible merges can be a very slow process. However, when merging two clusters, the contingency table can be easily updated by summing the appropriate two rows (or columns). Therefore, updating the contingency table scales as at most $O(K)$ and computing the NMI scales as less than $O(K^2)$. Since we have fewer than $\binom{K}{2} = O(K^2)$ possible merges, identifying the best merge at each step is at most $O(R \times K^4)$. There are at most $R \times K$ merges. So, overall, Dune scales as $O(R^2 \times K^5)$.

On the other hand, if the contingency table were recomputed at each step, or if the merging criterion did not rely on contingency tables but scaled as $O(n)$, the algorithm would scale as $O(R^2 \times (n + K^2) \times K^3)$. In practice, $K \ll n$. Indeed, Svensson and Beltrame [1] find that, to a first approximation, $K = O(\log(n))$. Thus, using the trick of merging based on contingency tables, we go from $O(R^2 \times n(\log(n)^3)$ to $O(R^2 \times (\log(n)^5)$. For large datasets with $n = O(10^6)$, this translates in practice to a 100-fold acceleration. Using merging criteria based on contingency tables makes Dune scalable to such large datasets and hence justifies our choice of the ARI or NMI.

Computational scalability using a greedy search. Dune only tries to merge two clusters at a time in just one of the partitions. This is an obviously greedy approach. To understand why such an approach is necessary, let us rephrase the problem Dune is trying to solve: Given a set of R clustering labels, maximize the average NMI by merging any set of clusters in one or more partitions. The general solution to this problem is to merge all clusters for all partitions. This leads to an average NMI of 1, but this degenerate solution is of no practical interest.

To eliminate this issue, an option would be add a penalization term Λ that increases as more merging occurs, such that the function to maximize becomes $\overline{\mathrm{NMI}}(\mathbf{P}_1, \ldots, \mathbf{P}_R) - \Lambda(k_1, \ldots, k_R)$. This becomes a discrete optimization problem and we will show that the naive enumeration of all possible solutions is not feasible.

Solving by enumeration requires identifying all possible merges of any combination of clusters in all partitions at once. The number of possible merges in a partition P_r is the number of possible ways to group clusters together. This can be redefined as the total number of possible ways to partition

the set of clusters, that is, the Bell number $B(k_r)$ [36]. Then, since we have R partitions, we have $\Pi_{r=1}^{R} B(k_r)$ possible merging combinations. Computing the change in NMI resulting from such a merge still scales as $O(K^2)$, relying on the trick from the previous paragraph. Overall, switching from a greedy to a full-enumeration search means scaling from $O(R^2 \times K^5)$ to $O(K^2 \times \prod_{r=1}^{R} B(k_r))$. We can use the following inequality to have a lower bound on the growth rate of the Bell number: $\forall k, B(k+1) \geq (\frac{k}{2})^{k/4}$. Therefore, the growth of the Bell number is more than exponentially larger than K^3. Fully enumerating all possibilities is not feasible in practice and the solution of the penalized problem is not easily found. Since, in practice, the greedy approach of Dune finds an appropriate balance between improving clustering concordance and merging all clusters in reasonable time, we select this approach for our implementation.

S-1.4.1 Theoretical guarantees

As noted before, the true global optimum of Dune's objective function corresponds to merging all clusters which is non-informative. Over all simulations and real datasets, Dune never merges a partition entirely: the greedy search seems to only ever find local maxima, which is what is desired. Pathological cases may exists where this is not the case, and as such, our implementation of the algorithm will issue a warning when one or more partitions are fully merged, to make sure that users are aware of this fact.

However, we can still consider settings where the full merge will never happen. Consider a case where we have R partitions such that all partitions identify perfectly a common higher-level organization, i.e. all clusters of all partitions can be grouped into a common set. Using the formalism from the method section, this means that we have another partition $\mathcal{A}_1, \ldots, A_m$ such that: $\forall r \in \{1, \ldots, R\}, \forall k \in \{1, \ldots k_r\}, \exists! a \in \{1, \ldots, m\}$ such that $\mathcal{C}_k^{(r)} \subset A_a$.

In this case, we can prove that two clusters from any partition that are not included in the same group will never be merged since the NMI improvement is negative. Without loss of generality, we can try to merge two clusters from $\mathbf{P}_1$, $\mathcal{C}_1^{(1)}$ and $\mathcal{C}_2^{(1)}$ such that $\mathcal{C}_1^{(1)} \subset A_1$ and $\mathcal{C}_2^{(1)} \subset A_2$ and we look at how the NMI with the second partition $\mathbf{P}_2$ evolves. We use the notation from the contingency table S1.

$$
\begin{aligned}
NMI(\mathbf{P}_1, \mathbf{P}_2) =& \frac{\mathbf{H}(\mathbf{P}_1) - \mathbf{H}(\mathbf{P}_1|\mathbf{P}_2)}{\mathbf{H}(\mathbf{P}_1) + \mathbf{H}(\mathbf{P}_2)} \\
NMI(\mathbf{P}_1^{1\cup 2}, \mathbf{P}_2) =& \frac{\mathbf{H}(\mathbf{P}_1) - \mathbf{\Delta H}_1 - \mathbf{H}(\mathbf{P}_1|\mathbf{P}_2) + \mathbf{\Delta H}_{1,2}}{\mathbf{H}(\mathbf{P}_1) - \mathbf{\Delta H}_1 + \mathbf{H}(\mathbf{P}_2)} && \text{with} \\
\mathbf{\Delta H}_1 =& - a_1 \log \frac{a_1}{a_1 + a_2} - a_2 \log \frac{a_2}{a_1 + a_2} && \text{and} \\
\mathbf{\Delta H}_{1,2} =& - \sum_{j=1}^{k_2} \left(n_{1,j} \log \frac{n_{1,j}}{n_{1,j} + n_{2,j}} + n_{2,j} \log \frac{n_{2,j}}{n_{1,j} + n_{2,j}} \right)
\end{aligned}
$$

For a given cluster $\mathcal{C}_j^{(2)}$, we have three possibilities:

$$
\begin{aligned}
\mathcal{C}_j^{(2)} \subset A_1 \implies & & n_{2,j} = 0 \\
\implies & n_{1,j} \log \frac{n_{1,j}}{n_{1,j} + n_{2,j}} + n_{2,j} \log \frac{n_{2,j}}{n_{1,j} + n_{2,j}} &= n_{1,j} \log \frac{n_{1,j}}{n_{1,j}} + 0 = 0 \\
\mathcal{C}_j^{(2)} \subset A_2 \implies & & n_{1,j} = 0 \\
\implies & n_{1,j} \log \frac{n_{1,j}}{n_{1,j} + n_{2,j}} + n_{2,j} \log \frac{n_{2,j}}{n_{1,j} + n_{2,j}} &= 0 + n_{2,j} \log \frac{n_{2,j}}{n_{2,j}} = 0 \\
\mathcal{C}_j^{(2)} \subset A_a, a \in \{3, \ldots, m\} \implies & & n_{1,j} = 0 \text{ and } n_{2,j} = 0 \\
\implies & n_{1,j} \log \frac{n_{1,j}}{n_{1,j} + n_{2,j}} + n_{2,j} \log \frac{n_{2,j}}{n_{1,j} + n_{2,j}} &= 0 + 0
\end{aligned}
$$

So we know that $\mathbf{\Delta H}1, 2 = 0$.

$$
\begin{aligned}
NMI(\mathbf{P}_1, \mathbf{P}_2) \leq NMI(\mathbf{P}_1^{1\cup 2}, \mathbf{P}_2) \iff & \frac{\mathbf{H}(\mathbf{P}_1) - \mathbf{H}(\mathbf{P}_1|\mathbf{P}_2)}{\mathbf{H}(\mathbf{P}_1) + \mathbf{H}(\mathbf{P}_2)} \leq \frac{\mathbf{H}(\mathbf{P}_1) - \mathbf{\Delta H}_1 - \mathbf{H}(\mathbf{P}_1|\mathbf{P}_2)}{\mathbf{H}(\mathbf{P}_1) - \mathbf{\Delta H}_1 + \mathbf{H}(\mathbf{P}_2)} \\
\iff & - \mathbf{\Delta H}_1 \times (\mathbf{H}(\mathbf{P}_1) - \mathbf{H}(\mathbf{P}_1|\mathbf{P}_2)) \leq -\mathbf{\Delta H}_1 \times (\mathbf{H}(\mathbf{P}_1) + \mathbf{H}(\mathbf{P}_2)) \\
\iff & \mathbf{\Delta H}_1 \times (\mathbf{H}(\mathbf{P}_2) + \mathbf{H}(\mathbf{P}_1|\mathbf{P}_2)) \leq 0
\end{aligned}
$$

However, since $\mathbf{\Delta H}_1 > 0$, $\mathbf{H}(\mathbf{P}_2) > 0$ and $\mathbf{H}(\mathbf{P}_1|\mathbf{P}_2) \leq 0$, the last statement is not possible. Therefore, $NMI(\mathbf{P}_1, \mathbf{P}_2) > NMI(\mathbf{P}_1^{1\cup 2}, \mathbf{P}_2)$, we do not merge $\mathcal{C}_1^{(1)}$ and $\mathcal{C}_2^{(1)}$. Therefore, **we never merge all clusters of a partition**.

S-1.5 Clustering algorithms for scRNA-Seq data

Any combination of clustering algorithms and associated tuning parameters, applied to an appropriate dataset, can produce a set of partitions that can be used as input to Dune. However, as our work was motivated by the classification of cells based on transcriptomic signatures, we will focus on this particular setting to benchmark Dune.

In the descriptions below, we use the notation from the original publications to describe the tuning parameters of each clustering method; the same notation may therefore correspond to different parameters depending on the algorithm.

SC3 [2] is a consensus clustering method that involves performing k-means clustering on different dimensionality reductions of the input dataset. A hierarchical clustering method is then applied to the resulting consensus matrix. The main tuning parameter is the number of clusters k, which is used both in k-means and to cut the hierarchical clustering tree. The method provides an estimate of the optimal value of this parameter, k_0, based on the number of eigenvalues of the centered and scaled distance matrix that are significantly different from 0 (see Kiselev et al. [2] for more details). For large datasets, there exists a hybrid version of the algorithm, where the full SC3 clustering method is run on only a fraction of the cells to identify the clusters and the rest of the cells are assigned to the clusters using a support vector machine (SVM) algorithm.

Seurat's clustering algorithm ($SEURAT, RRID : SCR\_007322$) has evolved over the different versions of the software; here, we focus on version 3 [3] (we specifically use version 3.1.1). The algorithm first reduces the dimension of the data by selecting the first p principal components (PCs) and then computes a k-nearest neighbor (k-NN) graph. After refining the graph, it groups cells using, as default, the Louvain algorithm [37]. The two main tuning parameters are the number of neighbors k used to build the k-NN graph and the resolution parameter for the Louvain algorithm.

Monocle's clustering algorithm has also changed and we focus on version 3 [4] (implemented in the Monocle3 package, although we keep the name Monocle for simplicity; we specifically use version 0.1.3). Monocle's clustering algorithm is similar to the one implemented in Seurat, with a few differences. After initial dimensionality reduction based on principal component analysis (PCA), Monocle performs another dimensionality reduction step using uniform manifold approximation and projection (UMAP) [38, 39] and relies on that representation to build the k-NN graph. It then clusters cells using, by default, the Leiden algorithm [40].

Resampling-based sequential ensemble clustering (RSEC) [8] is a consensus method over user-supplied clustering algorithms and their associated tuning parameters. In order to improve the stability and tightness of the clusters, it also provides the option to perform clustering on subsamples of the observations, as well as sequential clustering. However, in this paper, we mainly use RSEC for its final step of hierarchical merging, see section "Existing methods to merge clusters".

S-1.5.1 Tuning parameters

For each method, we only tune the main parameter. For Seurat, however, there are two main tuning parameters. The k parameter controls the number of neighbors used to build the k-NN graph, while the resolution parameter defines the neighborhood in the Louvain clustering algorithm. In practice, the k parameter has much less impact than the resolution parameter (see Figure S1). Moreover, depending on the value of the resolution, increasing k either increases or decreases the final number of clusters. Accordingly, we only consider changing the resolution parameter.

For ease and generality of notation, we will denote each method's main tuning parameter by θ and define θ such that increasing θ increases the number of clusters. Thus, for the methods described above, $\theta_{SC3} = k$, $\theta_{Seurat} = \text{Resolution}$, and $\theta_{Monocle} = -k$. Each combination $\Theta = \{\theta_{SC3}, \theta_{Seurat}, \theta_{Monocle}\}$ of the three parameters defines a set of partitions that serves as input for Dune.

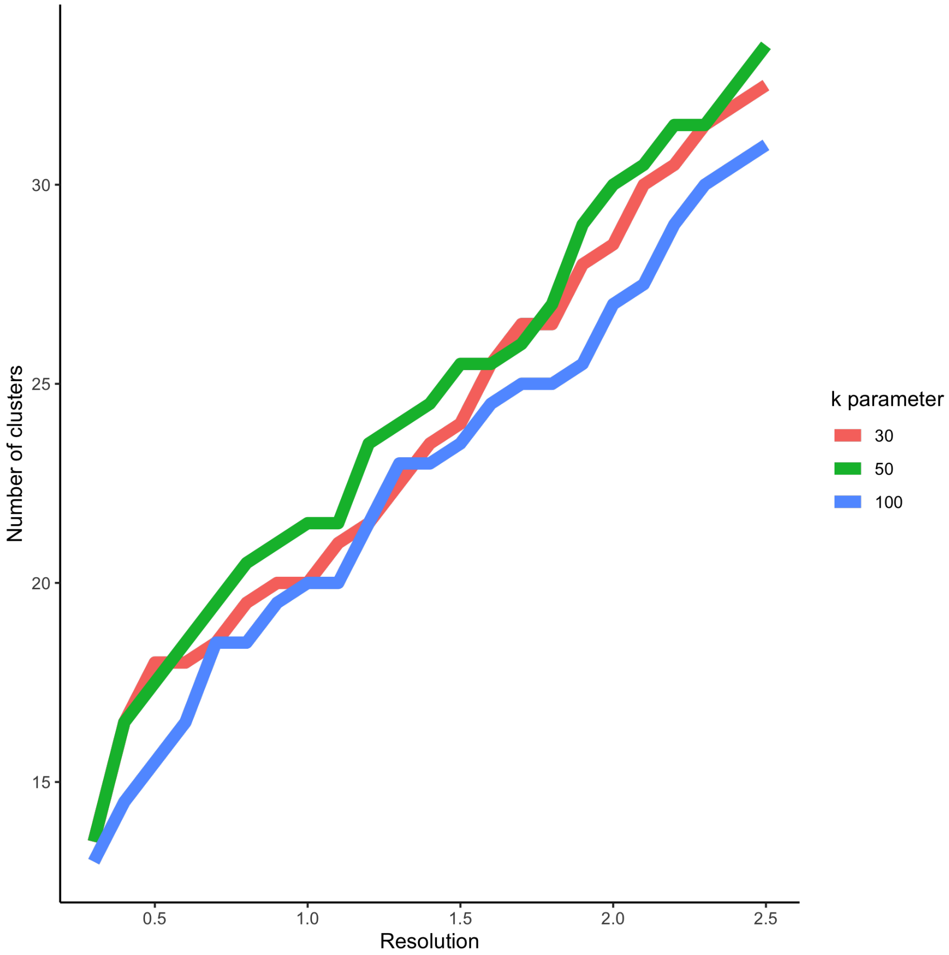

Figure S1: *Impact of Seurat's two main tuning parameters on the number of clusters.* The Seurat algorithm is run on the two AIBS mouse brain snRNA-Smart datasets, for a grid of tuning parameter values. The average number of clusters found in both datasets is then computed. For increasing values of the resolution parameter and fixed values of the k parameter, the number of clusters is always increasing. On the other hand, for increasing values of the k parameter and fixed values of the resolution parameter, the number of clusters can either increase or decrease. This can be seen in the fact that the curves are all increasing but intersect multiple times.

S-1.6 Cluster replicability using MetaNeighbor

We quantify the replicability of clusters across datasets by applying a modified version of unsupervised MetaNeighbor [18] ($MetaNeighbor, RRID : SCR\_016727$). MetaNeighbor requires as input two unnormalized datasets, two sets of cluster labels, and a set of highly variable genes. One of the datasets is treated as a test dataset, where all cluster labels are hidden, the other dataset is treated as a training dataset, whose labels are propagated to the test dataset through a cell-cell similarity network.

To identify replicating clusters, we computed replicability scores using the `MetaNeighborUS` function, with parameters `one-vs-best=TRUE` and `symmetric_output=FALSE`. Briefly, each pair of clusters (one in the training dataset, the other in the test dataset) receives a score based on how well the training cluster predicts the labels from the test cluster. Each cluster from the training dataset is then assigned a unique best matching cluster in the test set: the one it can predict the best. Finally, we compute a final score for each training set cluster by reducing the test set to the two best matching clusters using the original score. Therefore, the final score measures how well a training cluster predicts a specific test cluster compared to its closest neighbor. Then the role of the test and training datasets are reversed. Each cluster from the two datasets is therefore assigned a target in the other dataset, and an associated score.

A cluster is considered replicable if there is a cluster in the other dataset such that the clusters are reciprocal best hits with score > 0.6 both ways. See Crow et al. [18] for more details and a benchmark

of this method. Finally, the **replicability score of a clustering** is defined as the fraction of cells contained in replicable clusters. More specifically, for a comparison of two datasets, we enumerate replicable clusters in each dataset, then deduce the number of cells that are in replicable clusters, sum this number across datasets, and divide by the total number of cells.

We used MetaNeighbor's `variableGenes` procedure to select genes that are highly variable across all datasets. For computational cost reasons, the `variableGenes` procedure was applied to a random subset of 50,000 cells for datasets exceeding that size. However, the full datasets were used for the rest of the analysis. In the end, we obtained a set of 541 highly variable genes for the brain datasets and $2,147$ genes for the pancreas datasets.

S-1.7 Data analysis

Except when otherwise specified, all methods and algorithms were run with default parameters or, if no available default, with the parameters recommended in the vignette or tutorial.

Pre-processing. Count matrices were filtered to remove lowly-expressed genes with fewer than i reads in j cells. See Table S2 for values of i and j for each dataset.

As indicated below, we follow different normalization strategies before running Seurat and Monocle in order to obtain more diverse clustering results. This is appropriate, as the goal of the manuscript is not to compare different clustering methods, but rather different merging methods for given clustering results. The merging methods that Dune is compared to rely on only one clustering input; we therefore seek to benchmark merging methods using a variety of clustering inputs.

Seurat. Following the tutorial, we run `FindVariableFeatures` and `ScaleData` to normalize the data. Counts are log-transformed (adding 1 to avoid taking the log of zero) and normalized by sequencing depth. For the two pancreas datasets, batches are also normalized for using the `scaleData` function. Following principal component analysis, `FindNeighbors` and `FindClusters` are run for a number of neighbors k in $\{30, 50, 70\}$ and resolution θ from 0.3 to 2.5 in increments of 0.1.

SC3. The algorithm is run on a dataset normalized as above with the Seurat pipeline. The optimal value of k, k_0, is computed using the `sc3_estimate_k` function. The parameter θ is transformed to be $\theta_{SC3} = k - k_0$. SC3 is then run for values of θ ranging from -15 to $+15$ in increments of 1.

Monocle. zinbwave [8] is first used for normalization and dimensionality reduction on the filtered count data. For the two pancreas datasets, batches are included as model covariates. We select K, the number of reduced dimensions, based on a visual representation for each dataset, see Table S2. This first step of dimensionality reduction is followed by another using UMAP [39] with two dimensions. The resulting two-dimensional representation is then used to build the k-NN graph, with k ranging from 10 to 150 in increments of 10.

Dune. For a given set of values for $\Theta = \{\theta_{SC3}, \theta_{Seurat}, \theta_{Monocle}\}$, we get three sets of cluster labels that we can use as input to Dune.

Building the hierarchical tree. The output of each clustering method is used as input to RSEC's `makeDendogram` function. Then, we either cut the tree using R's `cutree` function (for the Dist merging method) or RSEC's `mergeClusters` function (for the DE merging method).

Cell type annotation. Each dataset is normalized as described in the Seurat paragraph. For each pair of datasets (mouse brain or human pancreas), one dataset is used as reference and the other as target for which cells are to be labeled. The reference dataset is labeled using the cluster labels either before or after merging with Dune, for all values of θ described above. Each cell in the target dataset is assigned a label and a score using the Seurat `TransferData` function. The average score across all cells is used to evaluate the quality of the annotation.

Producing "bad" clusters. For each value of the tuning parameters Θ, on the pancreas datasets, we add fully random inputs to Dune. That is, we create "bad" clusterings by randomly assigning each cell a number (or cluster label) between 1 and $(k_{SC3} + k_{Monocle} + k_{Seurat})/3$, where k denotes the number of clusters for a particular clustering algorithm. Since cells are assigned randomly, the size of the clusters will vary, but all clusters have the same expected size. To account for the stochastic nature of this procedure, we repeat this 10 times.

Downsampling. Downsampling the number of cells at the beginning of the analysis pipeline would affect both the quality of the input clusterings and the quality of the merging with Dune. As such, to test only the stability of Dune to the number of cells, we downsample the cells just before running Dune, that is, the clustering algorithms are run on the full dataset but only a subset of the dataset is used to decide which clusters to merge and in which order. Afterwards, cells that are not in the subsample are assigned to the merged clusters based on their original cluster labels. That is, if Clusters 1 and 2 are merged, all cells that were originally in Cluster 1 or Cluster 2, even those not selected in the downsampling and not used as input to Dune, are assigned to the merged cluster.

Table S2: *Parameters for pre-processing the datasets.* Each dataset is filtered such that we keep all genes with a least i reads in j samples. Then, zinbwave is run with K dimensions.

Dataset	i	j	K
**AIBS mouse brain scRNA-Smart**	50	50	30
**AIBS mouse brain snRNA-Smart**	50	50	14
**Baron**	5	5	10
**Segerstople**	5	5	20

S-1.8 Simulation study

Simulation study design. We generated two types of datasets using Splatter [12]. 'Simple' datasets had balanced numbers of cells per cluster, i.e., each cluster had $5,000/30 \approx 166$ cells, and the DE proportion (one-versus-all) was the same for every cluster. 'Hard' datasets had unbalanced designs, i.e., cells were randomly assigned to each cluster, and the DE proportion was sampled from a uniform distribution $\mathcal{U}[.75 * \texttt{DE}, 1.25 * \texttt{DE}]$. No batch effects were added, given that benchmarking of normalization procedures was of no interest for our purpose.

Simulations parameters for each dataset. Therefore, each dataset is defined by the DE parameter and a label 'hard' or 'simple'. Datasets with the same parameters are however only identical if the random seed is set to an identical value.

Table S3: *Simulation parameters.*

	DE	Type
Dataset 1	.1	'Simple'
Dataset 2	.1	'Simple'
Dataset 3	.05	'Simple'
Dataset 4	.1	'Hard'
Dataset 5	.05	'Hard'

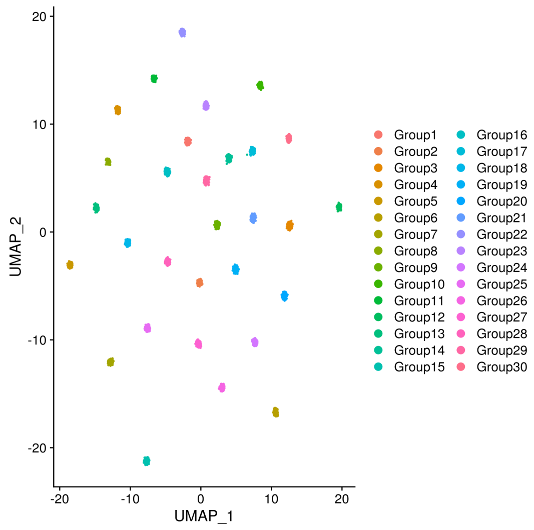

(a) Dataset 1

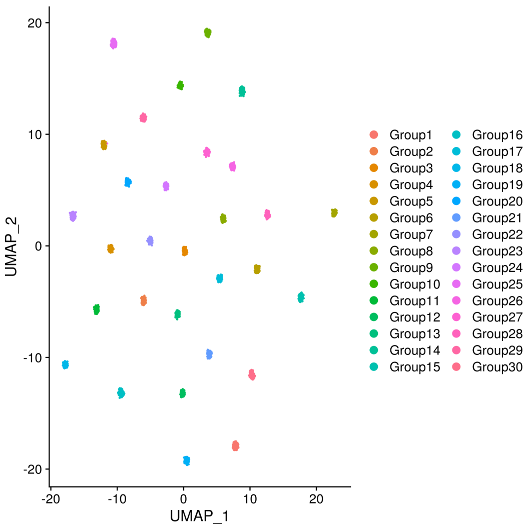

(b) Dataset 2

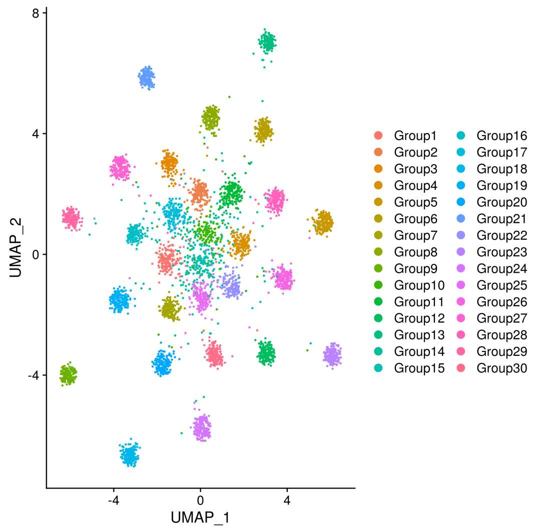

(c) Dataset 3

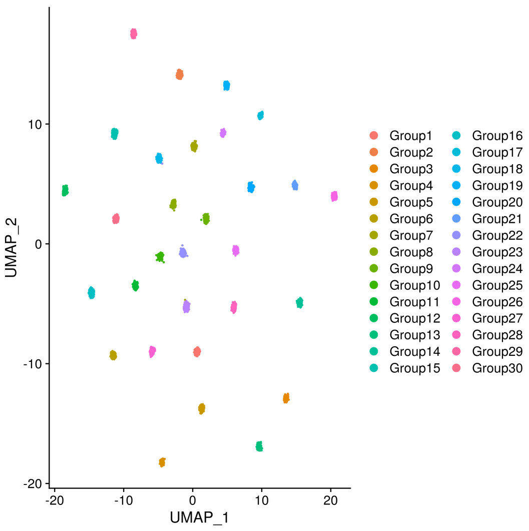

(d) Dataset 4

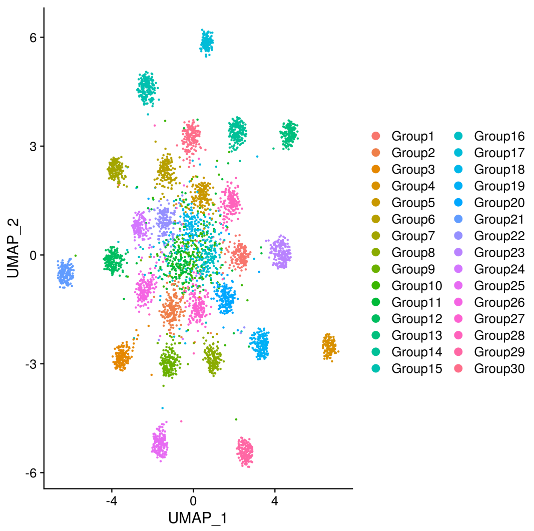

(e) Dataset 5

Figure S2: *Simulated datasets.* Two-dimensional representations of the simulated datasets using UMAP; plotting symbols for the cells are colored by the ground-truth cluster labels.

S-2 Supplementary results

S-2.1 Resolution-replicability trade-off

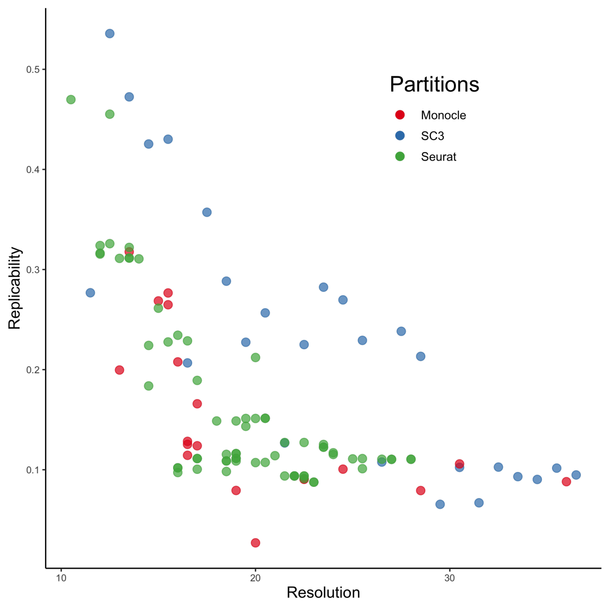

Figure S3: *Resolution-replicability trade-off for the Pancreas datasets.* Seurat, SC3, and Monocle are run on the two Pancreas datasets, as described in "Methods", for a wide range of tuning parameter values. Then, the MetaNeighbor method is used to compute replicability scores for the resulting clusters between these two datasets. A trade-off between replicability and resolution is visible.

S-2.2 Robustness analysis

S-2.2.1 Robustness on simulated datasets

Simulation settings. All simulations presented in the robustness analysis are for scenarios of type 'Simple' with a DE proportion of .1, just as Datasets 1 and 2 of the simulation benchmark.

Downsampling. We generated datasets of $n \in \{100, 200, 500, 1,000, 2,000, 5,000\}$ cells. On these datasets, we used SC3, tSNE+kMeans, and UMAP+kMeans (all with $k = 40$) as input to Dune. Then, the ARI with the ground truth was computed as merging occurred.

Varying tuning parameters. For the dataset with $n = 5,000$ cells generated above, we used SC3, tSNE+kMeans, and UMAP+kMeans as input to Dune. The tuning parameter was identical for all methods and varied between $k = 39$ and $k = 50$. Then, the ARI with the ground truth was computed as merging occurred.

Changing the input clusterings. For the dataset with $n = 5,000$ cells generated above, we used SC3, tSNE+kMeans, and UMAP+kMeans, with $k \in \{35, 40, 45\}$. We randomly sampled a set of R (between 2 and 9) clustering inputs among the 9 methods. This process was repeated at least 5 times for each value of R, or until all possible combinations were selected if $\binom{9}{R} < 5$. Then, the ARI with the ground truth was computed as merging occurred. To facilitate presentation, we take the average over all repetitions for a given clustering method with a given tuning parameter value and given value of R.

S-2.2.2 Robustness on real datasets

Robustness to poor clustering inputs. Since Dune takes as input the results from clustering algorithms, its results depend on the quality of the clusterings produced by these algorithms. In general, Dune will not be able to produce good clusters when merging only clusters that capture no underlying biological signal. However, we showed that Dune is robust to a mix of "good" clustering inputs and "bad" clustering inputs. We used as "good" inputs the results of SC3, Seurat, and Monocle

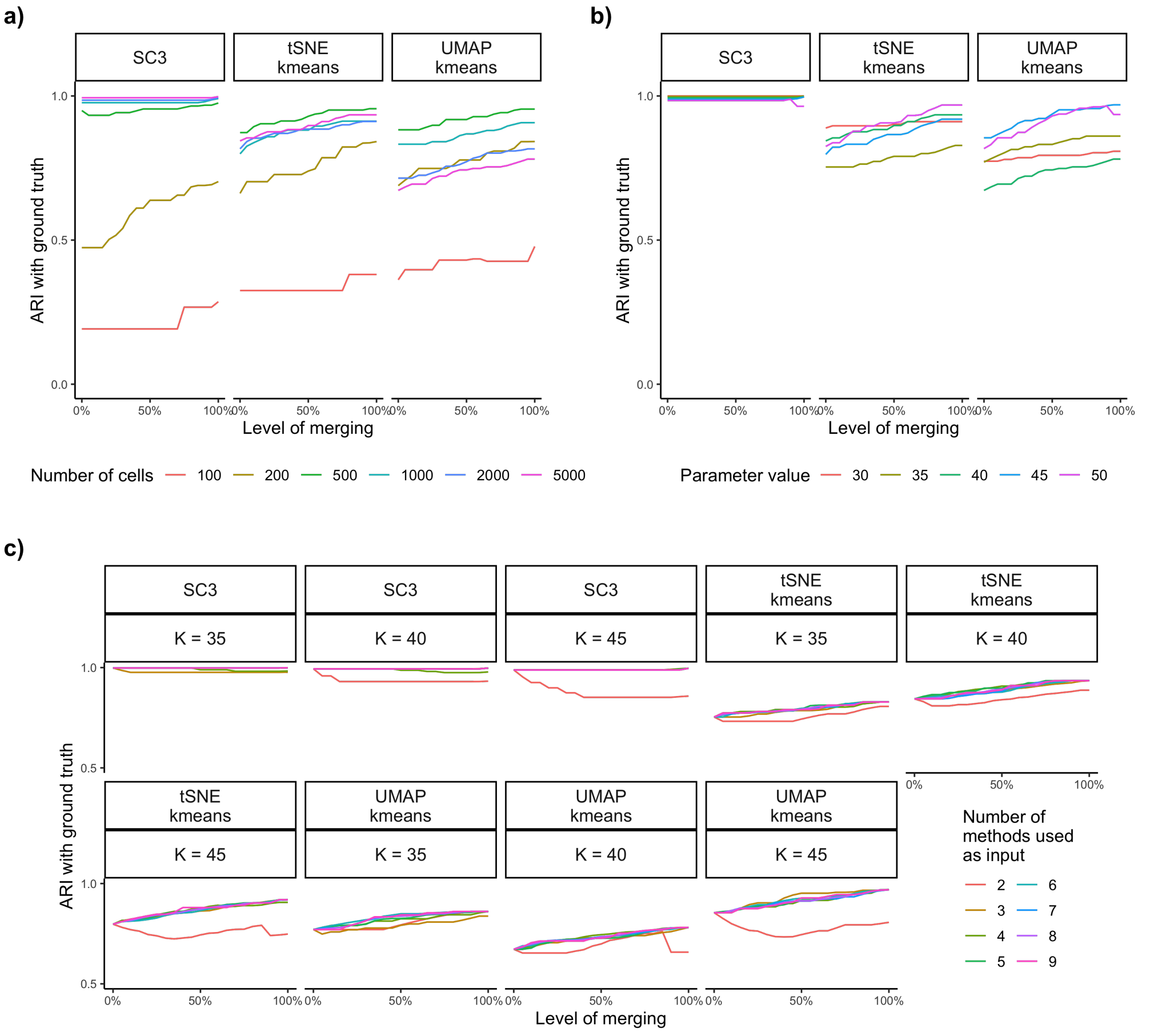

Figure S4: *Dune robustness analysis on simulated datasets.* 'Simple' simulated datasets with a DE proportion of .1 are generated. Clusterings from SC3, tSNE+kMeans, and UMAP+kMeans are used as inputs to Dune. Panel **a.** There are $R = 3$ inputs, $k = 40$ for each clustering method, and the number of cells n varies from 100 to $5,000$. The quality of the final clusterings is stable to downsampling up to 90%. Panel **b.** Keeping $R = 3$ and $n = 500$, the tuning parameter is changed from $k = 30$ up to $k = 50$. Nearly all partitions are improved by Dune. If there is no over-partitioning ($k = 30$), Dune has very little impact on the quality of clusterings, i.e., there is little room for improvement. The initial ranking of methods is mostly left unchanged by Dune. Panel **c.** Keeping $n = 5,000$, the number of partitions used as input is changed, with partitions being randomly drawn from those described in panels **a.** and **b.**. Once $R \geq 3$, adding more clusterings as input has a limited effect on the results of Dune.

that are assumed to capture some common signal, and as "bad" inputs fully random clusters that will not have any commonality (see the "Methods, Data analysis" section). Then, the replicability of the "good" clusterings was measured as merging happened and the AURC was computed and compared to the AURC when there were no "bad" inputs. As more and more "bad" clusters were added (Figure S4b), Dune still improved the replicability of the "good" clusters as it merged them, even when half of the clusters used as inputs were random. Hence, Dune can recover from very poor clustering inputs.

Robustness to sample size. We investigated how Dune handles datasets with an ever-smaller number of cells. To simulate such datasets, we downsampled the two pancreas datasets. Downsampling could affect both the quality of input clusters and the merging procedure of Dune. To disentangle these two effects, we downsampled the two human pancreas datasets after running SC3,

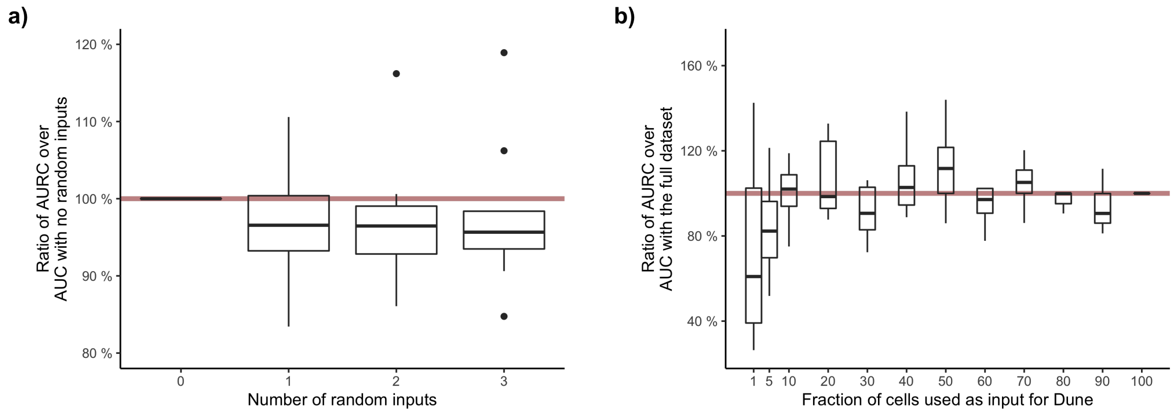

Figure S5: *Dune robustness analysis on real datasets.* Panel **a.** Adding an increasing number of random clustering inputs to Dune impacts only slightly the resolution-replicability area under the curve when merging the other correct clusters. Panel **b.** Likewise, Dune is stable to decreasing the number of input cells, as low as 10% of the original sample size.

Seurat, and Monocle, but before running Dune. We then measured how and whether merging still improved the cluster replicability by computing the AURC and constrasting it to its value without downsampling (see the "Methods, Data analysis" section for more details).

When the datasets were downsampled to between 90% and 10% of the original number of cells, Dune still correctly navigated the trade-off between resolution and replicability (Fig. S4c). Only when fewer than 10% of the cells were used (which amounts to datasets of fewer than 200 cells), did Dune's capacity to improve clustering replicability worsened noticeably. This demonstrates that the method is very stable to the number of cells.

S-2.3 Results for Dune with ARI merging

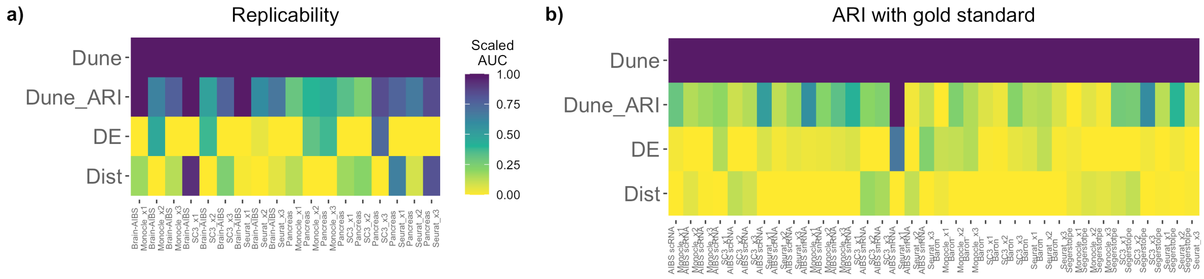

Figure S6: *Comparison of methods.* SC3, Seurat, and Monocle were run on all case studies datasets, for a wide range of tuning parameter values. Then, merging by Dune with ARI, Dune with NMI, and the two hierarchical procedures is evaluated using either replicability, measured via the MetaNeighbor method, or ARI with gold-standard labels. This yields 18 comparisons of AUC for replicabilty (**a.**) and 36 comparison of AUC for ARI with gold standard (**b.**). AUC values are displayed in the pseudocolor image, after being scaled to have a column mean of zero and column variance of 1. This was done to make AUC values comparable across datasets, clustering methods, and parameter values, since the AUC can have different scales across scenarios.

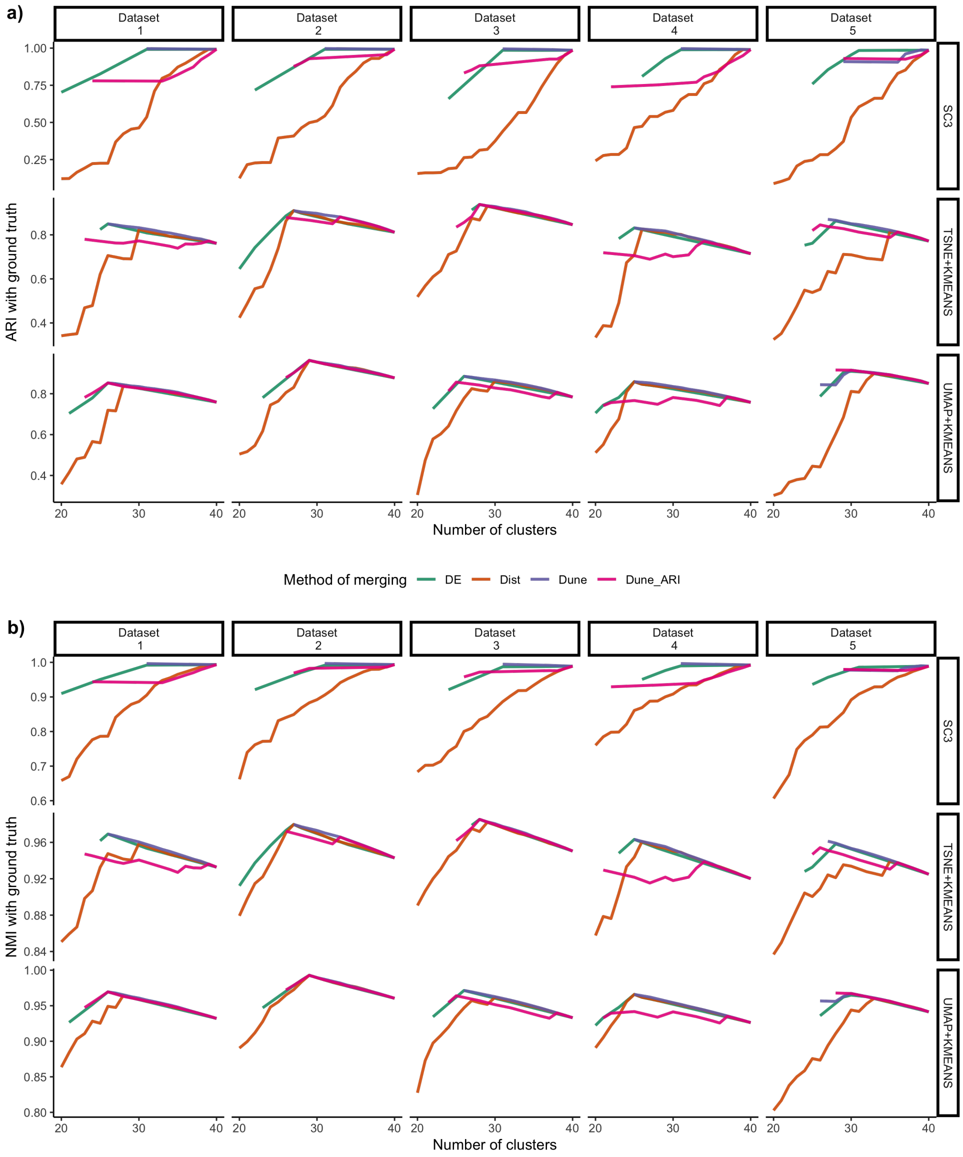

Figure S7: *Simulation results including the ARI-based version of* Dune*.* For each of the four merging methods, as merging occurs on a simulated dataset, the ARI (**a.**) or NMI (**b.**) with the ground truth is tracked as the number of clusters decreases. Dune with ARI performs either on-par or sub-par with the NMI-based version.

S-2.4 Keeping rare cell types

A potential drawback of Dune is inherent to its design. Since merging occurs, it is possible that rare cell types will be merged into larger groups. However, this should not be the case if that rare cell type is distinct enough such that all or most of the input clustering methods can clearly identify it. Here we see a balance between the distinctiveness and the size of the cell type.

To quantify this better, we look at the 4 real case studies, and we define a distinct cell type using the original labels as a cell type that represent less than 5% of the dataset. We remove the cell types with less than 5 cells, and those where the silhouette width (computed using euclidean distance in the reduced dimension space) is negative, since those represent mostly artifacts such as doublets.

Then, for each clustering method, we identify the cluster which maps the best to each cell type, using the Jaccard index and track that cluster are merging occurs. This results, across all 4 datasets, and each 3 values of Θ in 180 pairs of cell type / clusters. After running Dune, the cluster - potentially now merged with others - remains the best match for its cell type in 174 / 180 cases. Moreover, in 137 / 180 cases, the cluster is either not merged (126 cases), or merged such that it better match the cell type (9 cases). Therefore, overall, Dune is mostly able to preserve the small clusters.

We then estimates the remaining 43 cases in more details. First, those mostly happen in the **Segerstolpe** dataset, then in **Baron**, and then equally in the two mouse brain datasets. This is consistent with the fact that all values - ARI with gold standard, replicability - in the two pancreas datasets are lower than in the the two mouse brain datasets. Secondly, the initial Jaccard Index value is also higher in the 137 cases versus those 43 cases (.56 versus .42, p = .001 for a two-sample t-test). This means that Dune is more likely to merge the small cell types if they are poorly identified to start with. Finally, we consider all three inputs together. Our initial assumption was that merging would only occur zero or one clustering method finds that cell type. Therefore, we focus on the median Jaccard index of all three clustering method, for one dataset and one value of Θ. We compare this median value to the number of times the cluster remains the best hit with the same or a higher Jaccard Index (value between zero and three), which we name *hits*. Doing a partial correlation test using spearman correlation coefficient yields a coefficient of .41 and a p-value of .001. This shows that Dune is more likely to miss small cell types if at least two methods fail to identify it as input.

Dune workflow on the baron dataset

Hector Roux de Bézieux

03 November , 2020

Contents

1 Load data **1**

2 Pre-processing **2**

3 Creating inputs to Dune **4**
3.1 **SC3** 4
3.2 Seurat 5
3.3 Seurat with SCVI 6

4 Dune **7**
4.1 Running **Dune** 7
4.2 Vizualing the merging 7

5 Picking the final clustering result to use **8**
5.1 Manual selection 8
5.2 Selection based on sihouette 10
5.3 Comparing with the original labels 11

6 Runtimes **11**

7 Session **12**

References **14**

In this workflow, we will demonstrate a full full scRNA-Seq workflow using **Dune** on an example dataset. We rely on the the data from (Baron et al. 2016), a human pancreas dataset of 8569 samples. We will demonstrate how to generate various input clustering results, how to merge clusters using **Dune** and how to select the best output for use in downstream analysis. We will also monitor run times to show the impact of running **Dune** versus a workflow without it.

1 Load data

We rely on a pre-processed dataset where the count matrix has already been computed, using the **scRNAseq** R package. The dataset also contains the id of the human donor for each cell, which are used as batch labels. It also contains the cell labels assignements from the original publication. Note that, in that publication, cells were clustered using hierarchical clustering with a final manual merging step.

```
set.seed(19)
suppressPackageStartupMessages({
  library(SingleCellExperiment)
```

```r
  library(stringr)
  library(scRNAseq)
})
# Load pre-processed dataset
sce <- BaronPancreasData()
```

```r
# Filter very lowly expressed genes for computational practices.
filt <- rowSums(counts(sce) >= 2) >= 10
sce <- sce[filt, ]
print(sce)
```

```
## class: SingleCellExperiment
## dim: 12336 8569
## metadata(0):
## assays(1): counts
## rownames(12336): A1CF A2M ... ZZZ3 pk
## rowData names(0):
## colnames(8569): human1_lib1.final_cell_0001 human1_lib1.final_cell_0002
##   ... human4_lib3.final_cell_0700 human4_lib3.final_cell_0701
## colData names(2): donor label
## reducedDimNames(0):
## spikeNames(0):
## altExpNames(0):
```

2 Pre-processing

Before running clustering algorithms, we will rely on two normalization pipelines.

- The default pipeline of **Seurat** (Cao et al. 2019).

```r
suppressPackageStartupMessages({
  library(Seurat)
})
pre_process_time <- system.time({
  se <- CreateSeuratObject(counts = counts(sce),
                           min.cells = 0,
                           min.features = 0,
                           project = "de")
  se <- AddMetaData(se, as.data.frame(colData(sce)))
  se <- NormalizeData(se, verbose = FALSE)
  se <- FindVariableFeatures(se, selection.method = 'vst', nfeatures = 4000,
                             verbose = FALSE)
  se <- se[VariableFeatures(se), ]
  se <- ScaleData(object = se, vars.to.regress = c("nCount_RNA", "donor"))
  sce <- as.SingleCellExperiment(se)
})
```

```
## Regressing out nCount_RNA, donor
```

```
## Centering and scaling data matrix
```

- The **scvi** method (Lopez et al. 2018).

```r
# Note that this chunk of code is actually python code run from R. To learn how
# to use the reticulate package, please follow
```

```r
# https://rstudio.github.io/reticulate/index.html
suppressPackageStartupMessages({
  library(reticulate)
})
use_python("/usr/local/linux/anaconda3.7/bin/python3")
scvi <- import("scvi")
anndata <- import("anndata")
np <- import("numpy")
sc <- import("scanpy")
scvi_time <- system.time({
  scvi$settings$seed = 0L
  adata <- anndata$AnnData(X = as.sparse(t(counts(sce))),
                           obs = data.frame(cells = colnames(sce),
                                            batch = sce$donor))
  scvi$data$setup_anndata(adata, batch_key = "batch")
  model <- scvi$model$SCVI(adata)
  model$train(n_epochs = 100L, n_epochs_kl_warmup = 25L)
})
```

We can visualize the latent space produced by **scvi** using the labels from the original publication, and reducing the 10 dimensions of the latent space to 2 using t-SNE (van der Maaten and Hinton 2008, van der Maaten (2014), Krijthe (2015)).

```r
suppressPackageStartupMessages(library(scater))
reducedDim(sce, "scvi") <- model$get_latent_representation()
denoised <- t(model$get_normalized_expression(adata, library_size = 10e4))
dimnames(denoised) <- dimnames(counts(sce))
assay(sce, "denoised") <- log1p(denoised)
sce <- runTSNE(sce, dimred = "scvi")
plotTSNE(sce, colour_by = "label")
```

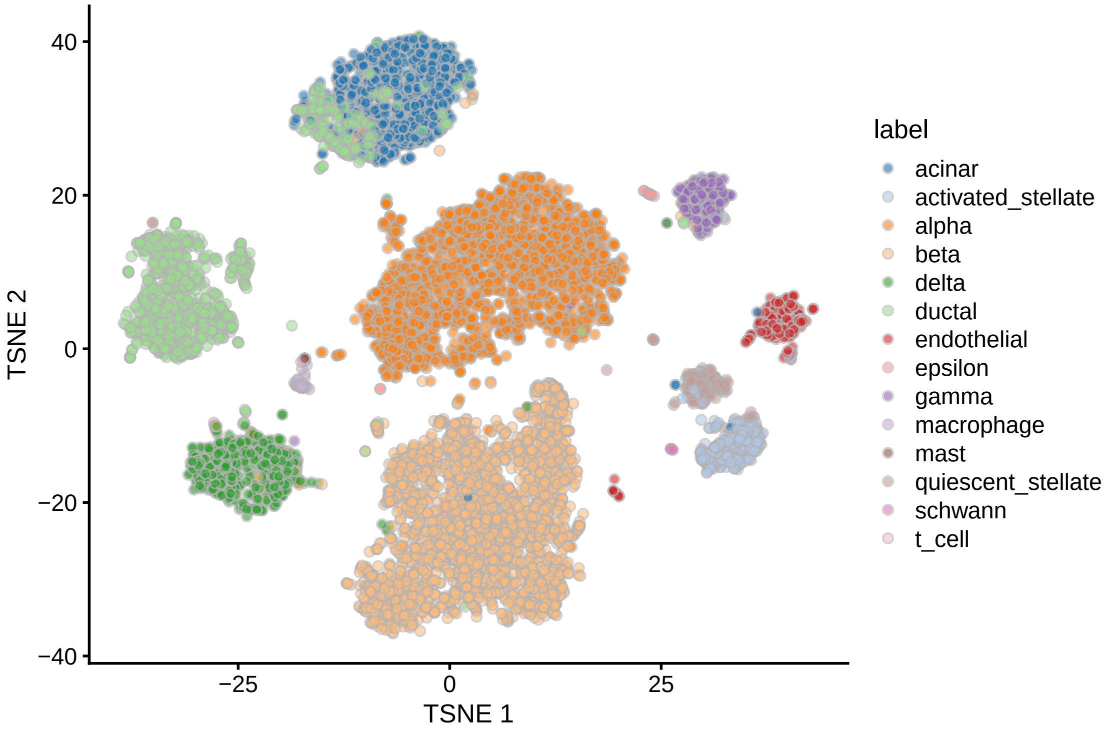

As we can see, **scvi** mostly produces a latent space that is consistent with the original labels. Note however that this is information that would not available while analyzing a new dataset. One would instead need to rely on known-marker genes.

3 Creating inputs to Dune

Dune takes as input a set of clustering results. We will generate a set of such results using a combination of clustering methods and normalization techniques:

- **SC3** (Kiselev et al. 2017) using as input the denoised count matrix from **scvi**.
- **Seurat** using as input the latent space from **scvi**.
- **Seurat** using as input the top pcs from the count matrix normalized using the **Seurat** pre-processing pipeline.

3.1 SC3

SC3 is a consensus method that takes as input a normalized count matrix and outputs a set of cluster labels. The **SC3** package provides a function to estimate the value of K, the exact number of clusters, which we will use.

Since the dataset has more than 5000 cells, **SC3** is automatically run in hybrid mode to lower runtime. However, the process can still be quite slow. The code below is run in the default mode. If you want it to run, we recommand seting `default=FALSE`.

```
suppressPackageStartupMessages(library(SC3))
default <- TRUE
sc3_time <- system.time({
  sce_sc3 <- sce
  logcounts(sce_sc3) <- assay(sce, "denoised")
  rowData(sce_sc3)$feature_symbol <- rownames(sce_sc3)
  counts(sce_sc3) <- as.matrix(counts(sce_sc3))
  logcounts(sce_sc3) <- as.matrix(logcounts(sce_sc3))
  sce_sc3 <- sc3_estimate_k(sce_sc3)
  K <- metadata(sce_sc3)$sc3$k_estimation
  # Note: with R >= 4.0, RStudio and Mac OS, this can fails.
  # A workaround is running
  # parallel:::setDefaultClusterOptions(setup_strategy = "sequential")
  if (default) {
    sce_sc3 <- sc3(sce_sc3, ks = K, n_cores = NCORES, rand_seed = 786907)
  } else {
    sce_sc3 <- sc3(sce_sc3, ks = K, n_cores = NCORES, rand_seed = 786907,
                   svm_num_cells = round(.1 * ncol(sce)))
  }
  sce_sc3 <- sc3_run_svm(sce_sc3, ks = K)
  sce$SC3 <- colData(sce_sc3)[, paste0("sc3_", K, "_clusters")] %>% as.factor()
})
```

```
plotTSNE(sce, colour_by = "SC3")
```

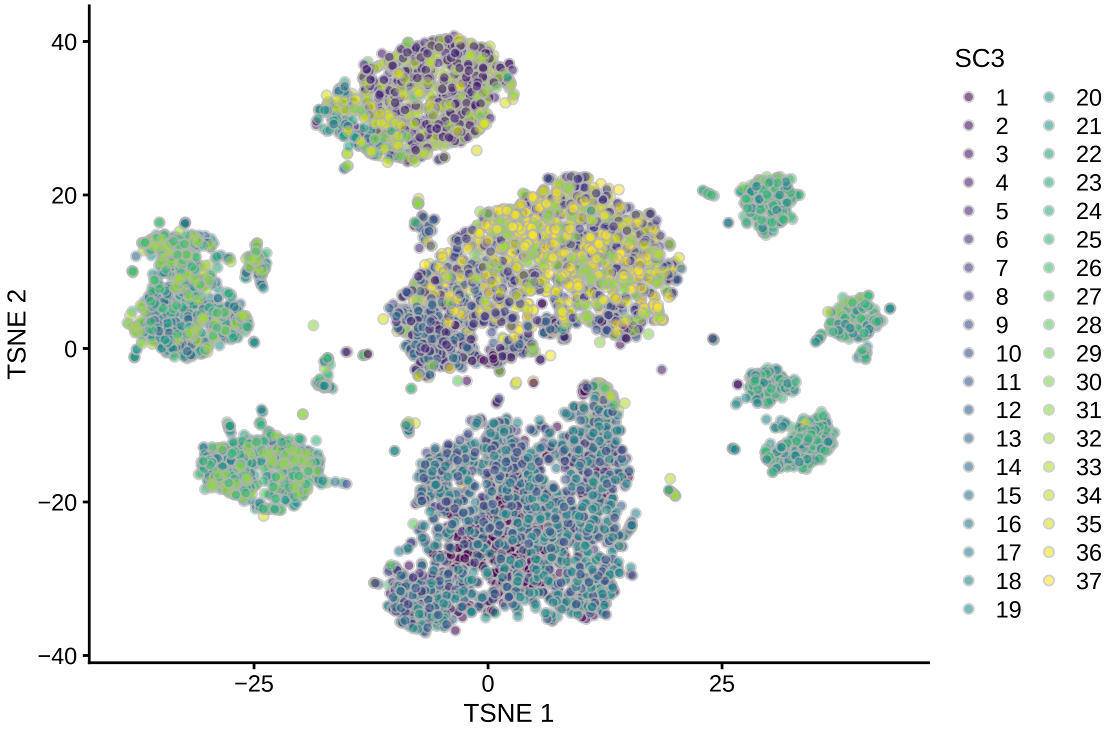

As we can see, **SC3** seems to overcluster the data, when compared either to the labels from the original publication, or to the reduced dimension representation. However, this is not a problem since **Dune** will work better on overclustered results.

3.2 Seurat

The second method we use is the clustering algorithm from the **Seurat** R package, which first construct a Shared Nearest Neighbor (SNN) Graph and then runs the Louvain algorithm on the graph to identify clusters. The SNN graph is build using a reduced dimension representation of the dataset. We first use the default, which is to use the top pcs from the normalized count matrix.

```
seurat_time <- system.time({
  se <- RunPCA(se, verbose = FALSE)
  se <- FindNeighbors(se, verbose = FALSE)
  se <- FindClusters(object = se, verbose = FALSE)
  sce$seurat <- Idents(se)
})
plotTSNE(sce, colour_by = "seurat")
```

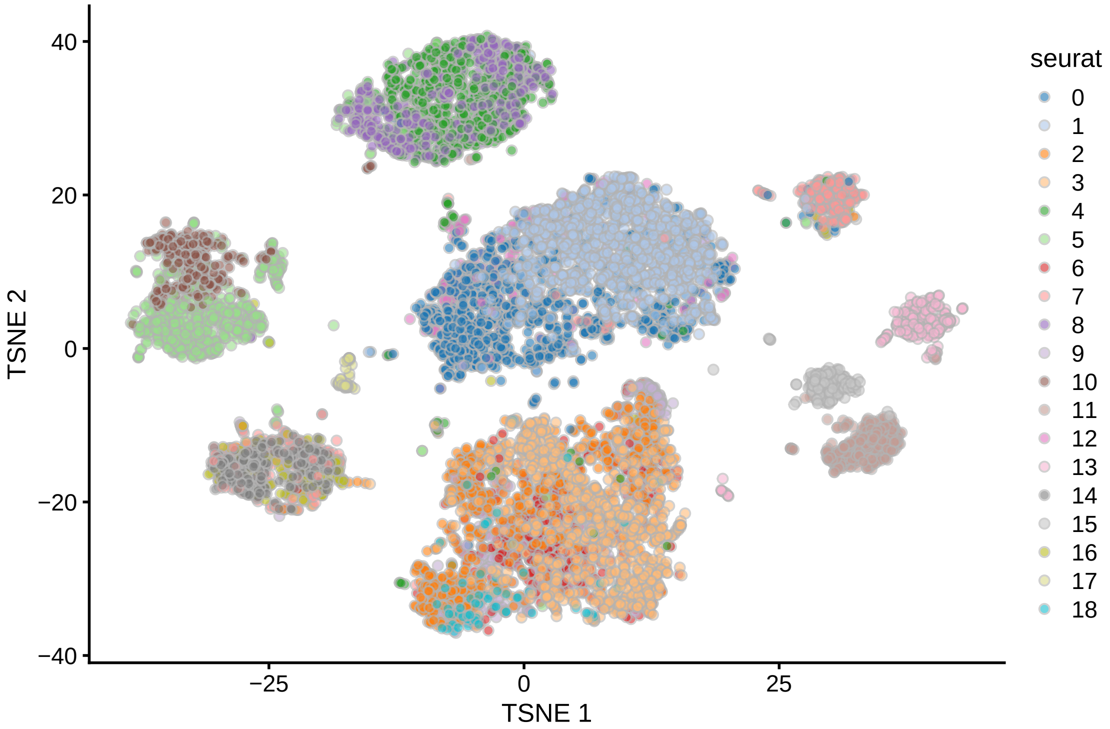

Seurat seems to perform better than **SC3** here but still seems to overpartition the data when run with the default parameters. Once again, it will not be a problem if used as input to **Dune**.

3.3 Seurat with SCVI

Finally, we run the **Seurat** clustering workflow, but instead of building the SNN using the top 10 pcs, we build it using the latent space from **scvi**.

```
seurat_scvi_time <- system.time({
  seu <- as.Seurat(x = sce, counts = "counts", data = "counts")
  seu <- FindNeighbors(seu, reduction = "scvi", verbose = FALSE)
  seu <- FindClusters(object = seu, verbose = FALSE)
  sce$seurat_scvi <- Idents(seu)
})
plotTSNE(sce, colour_by = "seurat_scvi")
```

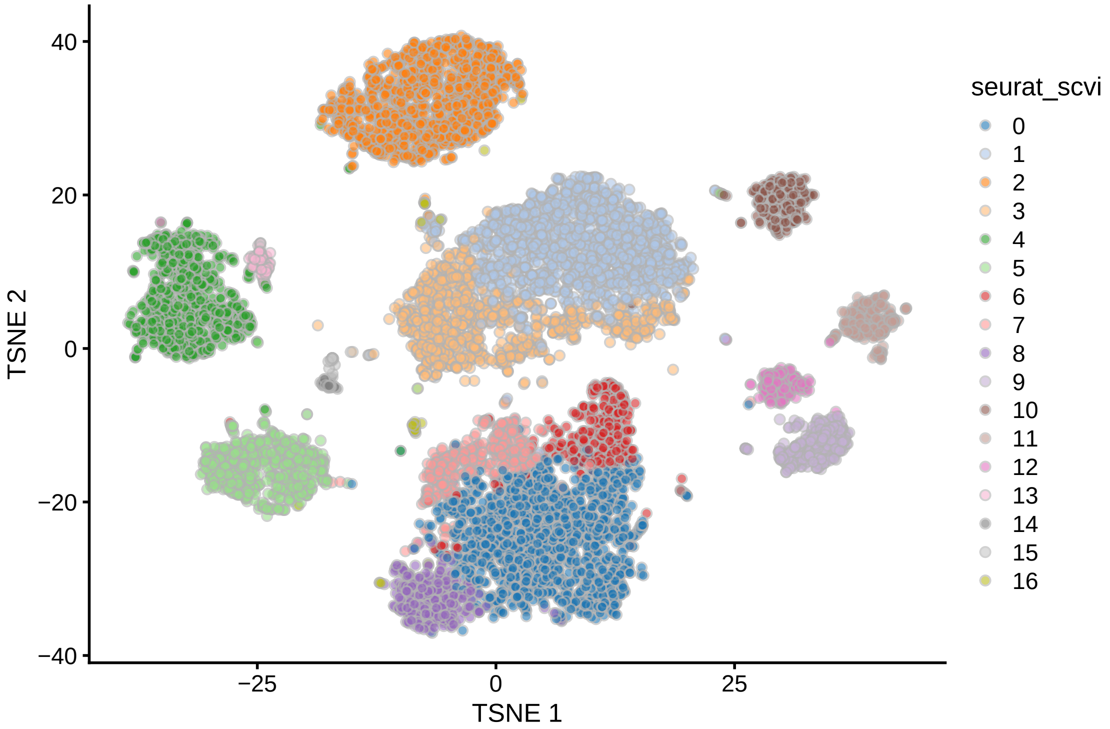

This seems to produce the best result, at least on the latent space of **scvi**, which is not suprising. It also better matches the labels from the original publication but still results in possible over-partition.

4 Dune

4.1 Running Dune

We can now run **Dune**, using the three clustering results as input. Since all clusterings seem to reflect over-partitioning of the data, **Dune** will identify the common underlying structure and polish all inputs, using the Normalized Mutual Information (NMI) as a merging criterion.

```
library(Dune)
df <- colData(sce)[, c("SC3", "seurat", "seurat_scvi")] %>%
  as.matrix()
dune_time <- system.time(
  merger <- Dune(clusMat = df, metric = "NMI")
)
colData(sce)[, c("SC3_final", "seurat_final", "seurat_scvi_final")] <-
  lapply(merger$currentMat, as.factor) %>%
    as.data.frame()
```

4.2 Vizualing the merging

We can first see how the number of clusters in each clustering set decreased as merging occurred, and how the mean NMI increased when merging.

```
NMItrend(merger)
```

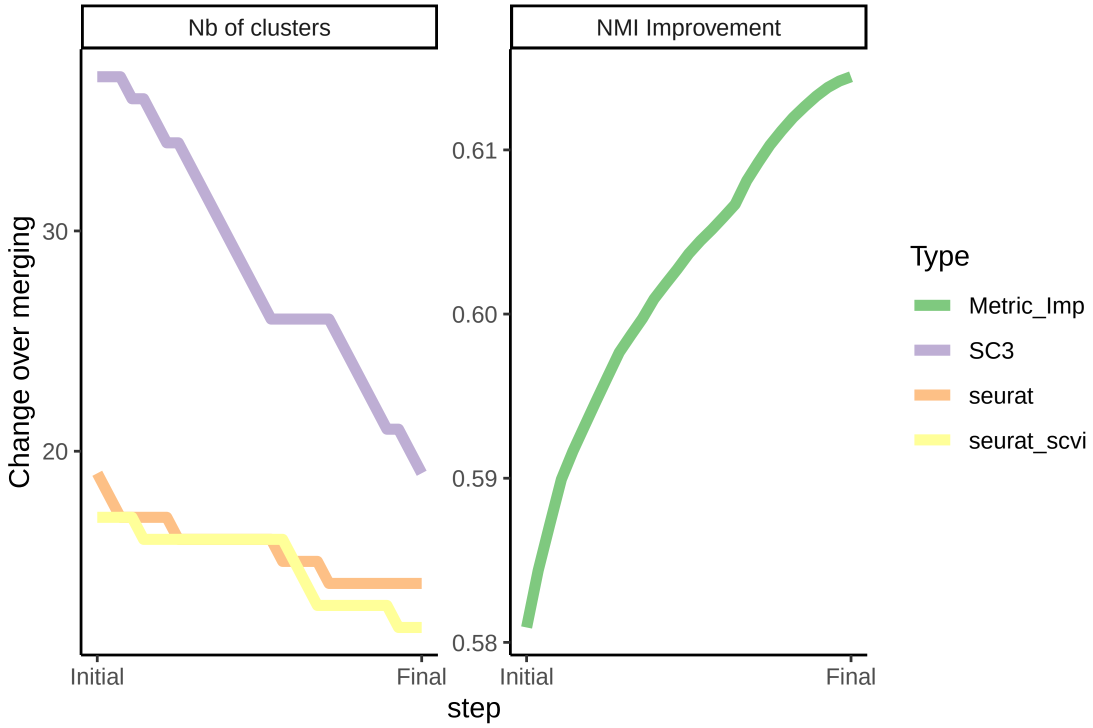

5 Picking the final clustering result to use

While **Dune** increases the concordance between the three sets of clusters, it does not pick one at the end. That choice remains up to the user. **Dune** does not seek to replace biological knowledge or other metrics used to rank clustering methods. Instead, it aims to improve all its inputs, and to lessen the impact of the selection of one set of clusters.

5.1 Manual selection

One common way to pick clustering results is still manual, using visualization.

```
plotTSNE(sce, colour_by = "seurat_final")
```

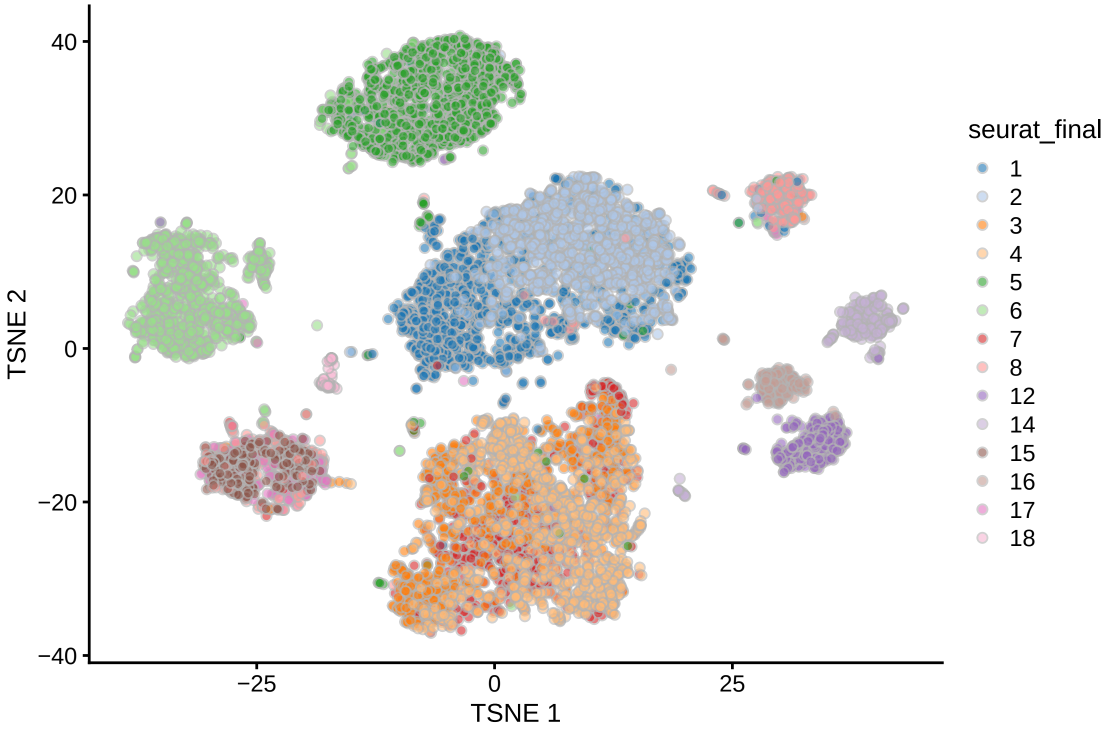


```r
plotTSNE(sce, colour_by = "seurat_scvi_final")
```

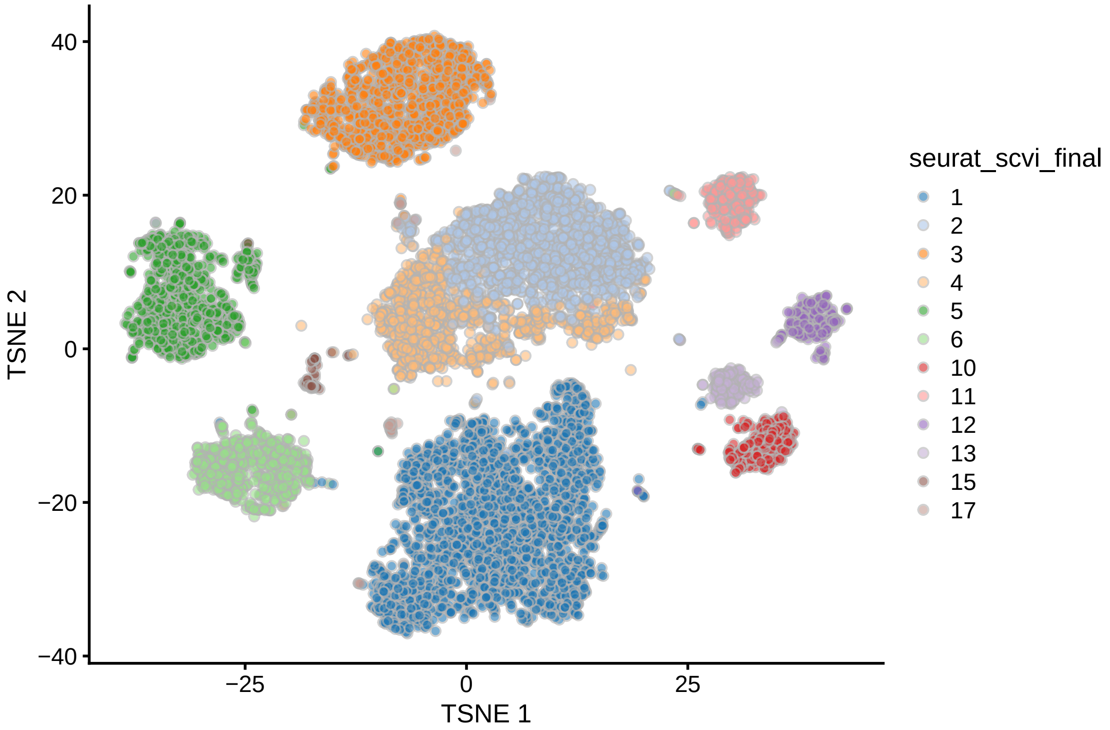


```r
plotTSNE(sce, colour_by = "SC3_final")
```

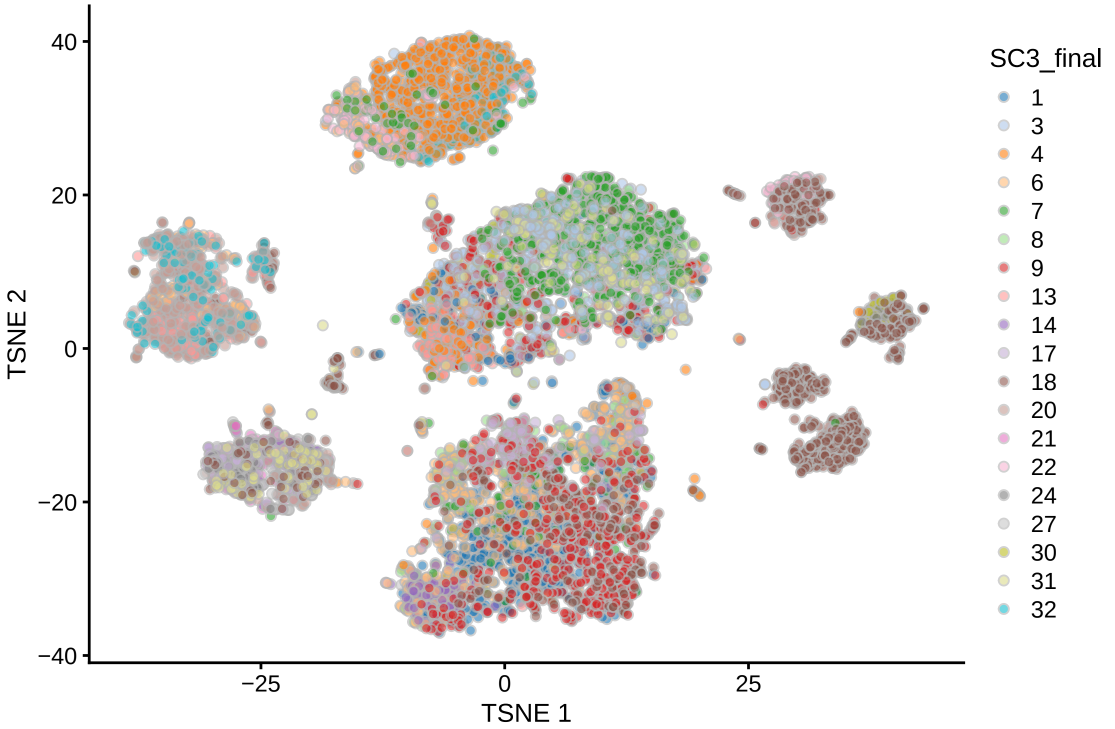

Here, we can see that all clustering results look more consistent with the low dimensionality representation. Moreover, it clearly looks like **Seurat** using the latent space from **scvi** produces better results.

5.2 Selection based on sihouette

To provide a more quantitative selection criterion, we can rely on the average silhouette width. This is a number between -1 and 1 that quantify the quality of clustering using the distance matrix between all cells. We compute the distance on the **scvi** latent space.

```
library(cluster)
dist_mat <- dist(as.matrix(reducedDim(sce, "scvi")))
sils_init <- lapply(merger$initialMat %>% as.data.frame, function(label){
  silhouette(label, dist = dist_mat)[,3] %>%  mean()
}) %>% unlist()
sils_init
```

```
##         SC3      seurat seurat_scvi
## -0.12061265  0.05173394  0.21896687
```

This confirm the visual impression: the cluster labels from **Seurat_scvi** are clearly better on this dataset than the others before merging with **Dune**.

```
sils_final <- lapply(merger$currentMat %>% as.data.frame, function(label){
  silhouette(label, dist = dist_mat)[,3] %>%  mean()
}) %>% unlist()
sils_final
```

```
##         SC3      seurat seurat_scvi
## -0.05884367  0.13027202  0.23961572
```

For all methods, the average silhouette information increased after merging with **Dune**. Even the best method, **Seurat_scvi**, is greatly improved by the merging. However, the ranking of methods is unchanged:

consistent with the visual representation, **Seurat** using the latent space from **scvi** clearly outperforms the other two. That is the one that should be used for downstream analysis such as trajectory inference, differential expression or cell type annotation.

5.3 Comparing with the original labels

This last step is not possible on a normal analysis of a new dataset. However, here, we can see how, running all methods using default, we recover cluster labels that match closely clusters from the original publication that had require manual merging using outside biological knowledge.

```
suppressPackageStartupMessages({
  library(aricode)
  library(mclust)
})
NMI(sce$label, sce$seurat_scvi_final) %>% round(2)
```

```
## [1] 0.83
```

```
adjustedRandIndex(sce$label, sce$seurat_scvi_final) %>% round(2)
```

```
## [1] 0.82
```

6 Runtimes

We can also compare the runtimes of all parts of the workflow. Running **SC3** in default mode is quite slow, followed by **scvi**. Running **Dune** itself is quite quick compared to other steps. Using **Dune** in a workflow increased total runtime but not by orders of magnitudes.

```
times <- c(pre_process_time[1],
           scvi_time[1],
           sc3_time[1],
           seurat_time[1],
           seurat_scvi_time[1],
           dune_time[1])
names(times) <- c("Seurat\npre-processing",
                  "SCVI",
                  "SC3",
                  "Seurat",
                  "Seurat\nafter SCVI",
                  "Dune")
df <- data.frame(times = times,
                 Name = factor(names(times), levels = names(times)))
ggplot(df, aes(x = Name, y = times, fill = Name)) +
  geom_col() +
  theme_classic() +
  labs(x = "Step", y = "Time (second)", fill = "Step") +
  scale_fill_brewer(palette = "Dark2") +
  theme(axis.text.x = element_text(angle = 90)) +
  scale_y_log10()
```

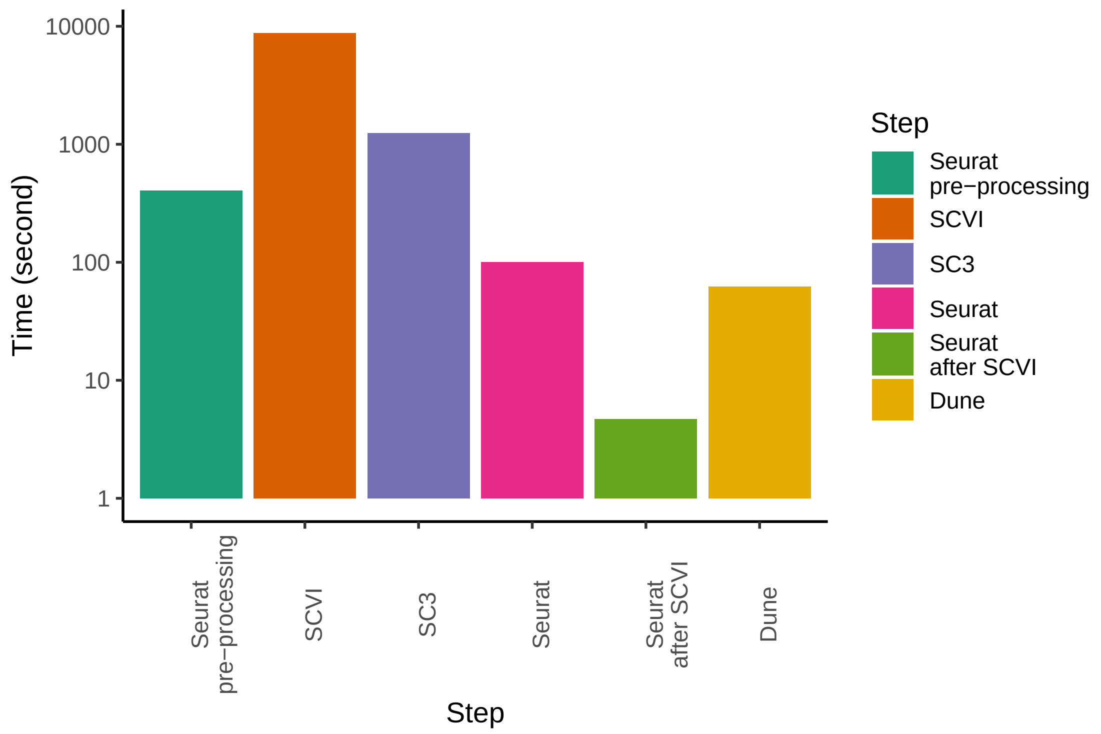

7 Session

```
sessionInfo()
```

```
## R version 3.6.1 (2019-07-05)
## Platform: x86_64-pc-linux-gnu (64-bit)
## Running under: Ubuntu 18.04.3 LTS
##
## Matrix products: default
## BLAS:   /usr/lib/x86_64-linux-gnu/openblas/libblas.so.3
## LAPACK: /usr/lib/x86_64-linux-gnu/libopenblasp-r0.2.20.so
##
## locale:
##  [1] LC_CTYPE=en_US.UTF-8       LC_NUMERIC=C
##  [3] LC_TIME=en_US.UTF-8        LC_COLLATE=en_US.UTF-8
##  [5] LC_MONETARY=en_US.UTF-8    LC_MESSAGES=en_US.UTF-8
##  [7] LC_PAPER=en_US.UTF-8       LC_NAME=C
##  [9] LC_ADDRESS=C               LC_TELEPHONE=C
## [11] LC_MEASUREMENT=en_US.UTF-8 LC_IDENTIFICATION=C
##
## attached base packages:
## [1] parallel  stats4    stats     graphics  grDevices utils     datasets
## [8] methods   base
##
## other attached packages:
##  [1] mclust_5.4.6                aricode_1.0.0
##  [3] cluster_2.1.0               Dune_1.1.0
##  [5] SC3_1.14.0                  scater_1.14.6
```

```
##  [7] ggplot2_3.3.1                Seurat_3.2.1
##  [9] scRNAseq_2.0.2               stringr_1.4.0
## [11] SingleCellExperiment_1.8.0  SummarizedExperiment_1.16.1
## [13] DelayedArray_0.12.3          BiocParallel_1.20.1
## [15] matrixStats_0.56.0           Biobase_2.46.0
## [17] GenomicRanges_1.38.0         GenomeInfoDb_1.22.1
## [19] IRanges_2.20.2               S4Vectors_0.24.4
## [21] BiocGenerics_0.32.0          knitr_1.28
## [23] rmarkdown_2.2                reticulate_1.13
## [25] SCF_3.6.1
##
## loaded via a namespace (and not attached):
##   [1] tidyselect_1.1.0              RSQLite_2.1.2
##   [3] AnnotationDbi_1.48.0          htmlwidgets_1.5.1
##   [5] grid_3.6.1                    Rtsne_0.15
##   [7] munsell_0.5.0                 codetools_0.2-16
##   [9] ica_1.0-2                     future_1.17.0
##  [11] miniUI_0.1.1.1                withr_2.1.2
##  [13] colorspace_1.4-1              ROCR_1.0-11
##  [15] robustbase_0.93-5             tensor_1.5
##  [17] listenv_0.8.0                 labeling_0.3
##  [19] GenomeInfoDbData_1.2.2        polyclip_1.10-0
##  [21] farver_2.0.1                  bit64_0.9-7
##  [23] pheatmap_1.0.12               vctrs_0.3.0
##  [25] generics_0.0.2                xfun_0.14
##  [27] BiocFileCache_1.10.2          R6_2.4.0
##  [29] doParallel_1.0.15             ggbeeswarm_0.6.0
##  [31] rsvd_1.0.2                    bitops_1.0-6
##  [33] spatstat.utils_1.17-0         assertthat_0.2.1
##  [35] promises_1.1.0                scales_1.0.0
##  [37] beeswarm_0.2.3                gtable_0.3.0
##  [39] npsurv_0.4-0                  globals_0.12.5
##  [41] goftest_1.2-2                 rlang_0.4.6
##  [43] splines_3.6.1                 lazyeval_0.2.2
##  [45] BiocManager_1.30.10           yaml_2.2.0
##  [47] reshape2_1.4.3                abind_1.4-5
##  [49] httpuv_1.5.3.1                tools_3.6.1
##  [51] ellipsis_0.3.1                RColorBrewer_1.1-2
##  [53] ggridges_0.5.1                Rcpp_1.0.5
##  [55] plyr_1.8.4                    progress_1.2.2
##  [57] zlibbioc_1.32.0               purrr_0.3.4
##  [59] RCurl_1.95-4.12               prettyunits_1.0.2
##  [61] rpart_4.1-15                  deldir_0.1-23
##  [63] pbapply_1.4-2                 viridis_0.5.1
##  [65] cowplot_1.0.0                 zoo_1.8-6
##  [67] ggrepel_0.8.1                 magrittr_1.5
##  [69] data.table_1.12.8             lmtest_0.9-37
##  [71] RANN_2.6.1                    mvtnorm_1.0-11
##  [73] fitdistrplus_1.0-14           hms_0.5.3
##  [75] patchwork_1.0.0               lsei_1.2-0
##  [77] mime_0.7                      evaluate_0.14
##  [79] xtable_1.8-4                  gridExtra_2.3
##  [81] compiler_3.6.1                tibble_2.1.3
##  [83] KernSmooth_2.23-15            crayon_1.3.4
```

```
## [85] htmltools_0.4.0           mgcv_1.8-28
## [87] pcaPP_1.9-73              later_1.0.0
## [89] tidyr_1.1.0               rrcov_1.4-7
## [91] DBI_1.0.0                 tweenr_1.0.1
## [93] ExperimentHub_1.12.0      WriteXLS_5.0.0
## [95] dbplyr_1.4.2              MASS_7.3-51.4
## [97] rappdirs_0.3.1            Matrix_1.2-17
## [99] igraph_1.2.4.1            pkgconfig_2.0.3
## [101] registry_0.5-1           plotly_4.9.0
## [103] foreach_1.4.7            vipor_0.4.5
## [105] rngtools_1.4             pkgmaker_0.27
## [107] XVector_0.26.0           bibtex_0.4.2
## [109] doRNG_1.7.1              digest_0.6.25
## [111] sctransform_0.2.0        RcppAnnoy_0.0.16
## [113] spatstat.data_1.4-3      leiden_0.3.3
## [115] uwot_0.1.8               DelayedMatrixStats_1.8.0
## [117] curl_4.3                 shiny_1.4.0.2
## [119] lifecycle_0.2.0          nlme_3.1-140
## [121] jsonlite_1.6.1           BiocNeighbors_1.4.2
## [123] viridisLite_0.3.0        pillar_1.4.2
## [125] lattice_0.20-38          fastmap_1.0.1
## [127] httr_1.4.1               DEoptimR_1.0-8
## [129] survival_2.44-1.1        interactiveDisplayBase_1.24.0
## [131] glue_1.4.1               gganimate_1.0.6
## [133] spatstat_1.64-1          png_0.1-7
## [135] iterators_1.0.12         BiocVersion_3.10.1
## [137] bit_1.1-14               class_7.3-15
## [139] stringi_1.4.3            blob_1.2.0
## [141] BiocSingular_1.2.2       AnnotationHub_2.18.0
## [143] memoise_1.1.0            dplyr_1.0.0
## [145] irlba_2.3.3              e1071_1.7-2
## [147] future.apply_1.3.0
```

References

Baron, Maayan, Adrian Veres, Samuel L. Wolock, Aubrey L. Faust, Renaud Gaujoux, Amedeo Vetere, Jennifer Hyoje Ryu, et al. 2016. "A Single-Cell Transcriptomic Map of the Human and Mouse Pancreas Reveals Inter- and Intra-cell Population Structure." *Cell Systems* 3 (4). Cell Press: 346–360.e4. doi:10.1016/j.cels.2016.08.011.

Cao, Junyue, Malte Spielmann, Xiaojie Qiu, Xingfan Huang, Daniel M. Ibrahim, Andrew J. Hill, Fan Zhang, et al. 2019. "The single-cell transcriptional landscape of mammalian organogenesis." *Nature* 566 (7745). Nature Publishing Group: 496–502. doi:10.1038/s41586-019-0969-x.

Kiselev, Vladimir Yu, Kristina Kirschner, Michael T Schaub, Tallulah Andrews, Andrew Yiu, Tamir Chandra, Kedar N Natarajan, et al. 2017. "SC3: Consensus clustering of single-cell RNA-seq data." *Nature Methods* 14 (5). Nature Publishing Group: 483–86. doi:10.1038/nmeth.4236.

Krijthe, Jesse H. 2015. *Rtsne: T-Distributed Stochastic Neighbor Embedding Using Barnes-Hut Implementation*. https://github.com/jkrijthe/Rtsne.

Lopez, Romain, Jeffrey Regier, Michael B. Cole, Michael I. Jordan, and Nir Yosef. 2018. "Deep generative modeling for single-cell transcriptomics." *Nature Methods* 15 (12). Nature Publishing Group: 1053–8.

doi:10.1038/s41592-018-0229-2.

van der Maaten, L.J.P. 2014. “Accelerating T-Sne Using Tree-Based Algorithms.” *Journal of Machine Learning Research* 15: 3221–45.

van der Maaten, L.J.P., and G.E. Hinton. 2008. “Visualizing High-Dimensional Data Using T-Sne.” *Journal of Machine Learning Research* 9: 2579–2605.
